# Proteomic and network analysis of human serum albuminome by integrated use of quick crosslinking and two-step precipitation

**DOI:** 10.1038/s41598-017-09563-w

**Published:** 2017-08-29

**Authors:** Zhao Liu, Shuiming Li, Haiyang Wang, Min Tang, Mi Zhou, Jia Yu, Shunjie Bai, Pengfei Li, Jian Zhou, Peng Xie

**Affiliations:** 1 Department of Neurology, the First Affiliated Hospital of Chongqing Medical University, Chongqing, 400016 China; 2 Institute of Neuroscience and the Collaborative Innovation Center for Brain Science, Chongqing Medical University, Chongqing, 400016 China; 3 Chongqing Key Laboratory of Neurobiology, Chongqing, 400016 China; 4 Shenzhen Key Laboratory of Microbiology and Gene Engineering, Shenzhen University, Shenzhen, 518060 China; 5 South Australian Health and Medical Research Institute (SAHMRI), North Terrace, Adelaide, SA 5000 Australia

## Abstract

Affinity- and chemical-based methods are usually employed to prepare human serum albuminome; however, these methods remain technically challenging. Herein, we report the development of a two-step precipitation (TSP) method by combined use of polyethylene glycol (PEG) and ethanol. PEG precipitation was newly applied to remove immunoglobulin G for albuminome preparation, which is simple, cost effective, efficient and compatible with downstream ethanol precipitation. Nonetheless, chemical extraction using TSP may disrupt weak and transient protein interactions with human serum albumin (HSA) leading to an incomplete albuminome. Accordingly, rapid fixation based on formaldehyde crosslinking (FC) was introduced into the TSP procedure. The developed FC-TSP method increased the number of identified proteins, probably by favouring real-time capture of weakly bound proteins in the albuminome. A total of 171 proteins excluding HSA were identified from the fraction obtained with FC-TSP. Further interaction network and cluster analyses revealed 125 HSA-interacting proteins and 14 highly-connected clusters. Compared with five previous studies, 55 new potential albuminome proteins including five direct and 50 indirect binders were only identified by our strategy and 12 were detected as common low-abundance proteins. Thus, this new strategy has the potential to effectively survey the human albuminome, especially low-abundance proteins of clinical interest.

## Introduction

Human serum albumin (HSA) is non-glycosylated and the most abundant circulating protein, and has an vital role in modulating blood volume by holding osmotic pressure^1, 2^. HSA is an electronegative, highly soluble protein with known transport and ligand-binding characteristics, enzyme activities and antioxidant properties. HSA has six binding sites and interacts with both exogenous and endogenous components, such as metal ions, drugs, peptide hormones and proteins^2–4^.

Research on plasma and serum-based proteomics over the last few decades has focused on depletion of HSA to discover potential protein biomarkers that are often found in low abundance^5^. However, the HSA-discarded fraction contains a large number of interesting molecules that reflect the pathophysiology of an individual’s symptoms^6^. In general, the HSA-binding peptides and proteins are collectively called the albuminome. This naturally occurring subproteome in blood is a valuable source for identifying disease-related biomarkers along with other pathological protein components^7, 8^. Such HSA-associated biomarkers may be beneficial for early detection of tumours^2, 9^. The potential usefulness of the albuminome suggests that detailed characterisation of this subproteome needs to be carried out.

To enrich serum HSA, antibody- or dye-based or liquid chromatography (LC) columns^9–11^, or chemical-based methods are commonly used^12, 13^. Affinity-based technologies have been evaluated and are effective at isolating HSA. However, these methods suffer from nonspecific protein/peptide adsorption to the column media and ligands, and carryover can arise if used for successive runs^12, 14–19^. For instance, in the immunoprecipitation-based method as reported by Zhu *et al*.^20^, a control is required for removing non-specific bindings to protein A + G agarose beads. Alternatively, the chemical ethanol precipitation method has been routinely utilized for isolating HSA^21^. Chemical fractionation can eliminate the issue of protein carryover in subsequent experiments, used for any sample volume, and is compatible with both two-dimensional gel electrophoresis and LC-tandem mass spectrometry (LC-MS/MS). This process was substantially modified by Fu *et al*.^13^ based on the classical Cohn fractionation schema^21^. Before ethanol precipitation, immunoglobulin G (IgG) as a common high-abundance protein in serum is depleted using a protein G affinity column prior to HSA purification. In this approach, the commercially available affinity column is a relatively expensive tool owing to the costly synthesis of affinity ligand-coupled polymers. Moreover, affinity columns have a limited capacity to partition larger sample volumes or quantities^14, 15, 18^. This affinity method has been shown to also unavoidably remove other serum proteins through non-specific binding to either the protein G molecule or to the Sepharose beads to which protein G is conjugated. Importantly, it is impossible to successively use the affinity column due to this non-specific adsorption^10, 16^. Thus, this method is relatively costly for multiple analyses of clinical samples.

Previously, we carried out proteomic and metabolomic analyses of human plasma to identify some potential biomarkers for the monitoring of major depressive disorder (MDD)^22–24^. Notably, our proteomic work focused on the HSA-depleted fraction from plasma samples of MDD patients^23^. However, in this disorder, the albuminome as an important subproteome may also be altered along with the disease state. Therefore, it prompted us to develop a practical and superior approach for future albuminome-related research. In the present study, an effective precipitation method to replace protein G-based immune depletion was designed by employing the readily available inexpensive reagent polyethylene glycol (PEG). The developed method combined PEG and ethanol precipitation for IgG removal followed by HSA enrichment. Upon chemical extraction using a two-step precipitation (TSP), we postulated that a fraction of the weak and transient protein interactions with HSA would dissociate to a small extent. Accordingly, quick fixation based on formaldehyde crosslinking (FC) was introduced into the TSP procedure for real-time and comprehensive profiling of the human serum albuminome.

## Materials and Methods

### Collection and delipidation of human serum samples

The study was approved by the Ethical Committee at Chongqing Medical University. According to the approved guidelines and regulations, human blood samples were collected at the Medical Examination Center of the First Affiliated Hospital of Chongqing Medical University from healthy individuals after written informed consent. Whole blood was collected into blood tubes without additives from six healthy individuals (three were male and three were female) aged 18–55 years, and centrifuged for 15 min at 3000 rpm. After centrifugation at 15,000 × *g* for 15 min, the lipids of the serum were discarded as previously described^13^. The resultant serum samples were pooled in equal volumes for subsequent experiments.

### FC-based quick fixation

Quick fixation of human serum samples was initially performed using formaldehyde according to the method described^20^. Briefly, solutions of 1, 5, 10, and 20% formaldehyde were prepared by diluting 40% stock solution with phosphate-buffered saline (pH 7.2–7.4). Then, 800 µL of the above diluted formaldehyde solution was added to 100 µL of serum samples. The solutions were gently mixed and incubated for various times at 4 °C. Then, a portion of a 4 M Tris quenching solution was immediately added and the mixture was incubated for 5 min. The cross-linking times were strictly controlled. A total of 400 µL quench solution was used for the 1–10% formaldehyde and 800 µL was used for the 20% formaldehyde reactions. Mixing formaldehyde and Tris before adding to the serum acted as the time zero control. The treated samples were mixed with 2 × gel loading buffer (250 mM Tris-HCl, pH 6.8, 2% sodium dodecyl sulfate [SDS] (w/v), 100 mM dithiothreitol [DTT], 20% glycerol (v/v), and bromophenol blue), and heated for 10 min at 65 °C before SDS-polyacrylamide gel electrophoresis (SDS-PAGE).

### PEG and ethanol precipitation

To the uncrosslinked and crosslinked samples, a 48% (w/v) PEG (PEG4000 or PEG6000) solution was added to a final concentration of 12% (w/v) PEG. The solution was incubated on ice for 30 min, followed by centrifugation at 12,000 × *g* for 15 min at 4 °C^25^. The PEG-soluble supernatant was mixed with various concentrations of ethanol and incubated at 4 °C for 1 h followed by centrifugation for 45 min at 16,000 × *g*. The ethanol-soluble supernatant (HSA-enriched fraction) was collected and then extracted with ice-cold acetone. The obtained proteins were stored until further analysis. For the uncrosslinked sample, 30 µL of serum (~2 mg proteins) was diluted with phosphate-buffered saline to 500 µL before adding PEG to provide an appropriate concentration. For the crosslinked sample, PEG was added directly after FC-based fixation.

### SDS-PAGE and immunoblotting

For SDS-PAGE analysis, the samples were dissolved in 1× gel-loading buffer. Unless otherwise specified, the uncrosslinked samples were boiled at 95 °C for 5 min, and the crosslinked samples were boiled for 20 min to reverse the formaldehyde crosslinking^26, 27^. After centrifugation at 14,000 × *g* for 15 min, the proteins in the supernatants were resolved in parallel lanes using SDS-PAGE gels and subsequently stained with Coomassie blue R-250. Meanwhile, immunoblotting was carried out according to our previously described procedures^28^. The primary antibodies used were anti-HSA (ab84348, 1:2000) and anti-human IgG (ab109489, 1:4000). The secondary antibody, horseradish peroxidase-conjugated anti-rabbit IgG (Bio-Rad, Hercules, CA, USA), was applied at 1:15,000. The densitometric analysis of the obtained images was performed using Quantity One software (Bio-Rad).

### Solution-based and gel-based sample preparation for MS detection

For solution-based sample preparation, protein pellets from the HSA-enriched fractions were solubilized in SDT buffer (4% SDS, 10 mM DTT, 150 mM Tris-HCl, pH 8.0). The uncrosslinked samples were boiled at 95 °C for 5 min and the crosslinked samples were boiled for 20 min. After centrifugation at 40,000 *g* for 15 min, protein concentrations were determined using a BCA protein assay kit (Pierce, USA). Subsequently, the proteins were tryptically digested by our previously used filter-aided sample preparation method^28, 29^. As previously reported^27^, formaldehyde-derived crosslinks can be preserved if samples are only incubated at 65 °C. In the present study, for gel-based sample preparation, the crosslinked samples were heated for 10 min at 65 °C and then separated with SDS-PAGE as described above^20^. The portion of the gel corresponding to HSA and its above protein bands was excised in two slices. The slices were subjected to trypsin digestion according to our previously described procedures^30, 31^. After in-solution and in-gel digestion, the resulting peptides were collected for MS detection.

### LC-MS/MS analysis

The tryptic peptides were analysed using a TripleTOF 5600 plus mass spectrometer (AB SCIEX) coupled with a splitless nanoLC-Ultra 2D plus system (Eksigent, Dublin, CA, USA). The Nanoflex system uses a desalting column C18 column (100 μm × 3 cm, 3 μm) and separation column (75 μm × 15 cm) packed with ChromXP C18 (3 μm, 120 Å). Samples were loaded on the trapping column at 2 μL/min and desalted by washing with a 100% mobile phase A (2% acetonitrile/0.1% formic acid/98% water) for 10 min. The peptide mixture was separated using a 120-min gradient at a flow rate of 300 nL/min. The gradient started with a 5% mobile phase B (98% acetonitrile/0.1% formic acid/2% water), and linearly progressed to 26% over 80 min and to 40% over 23 min, followed by an increase to 90% that was held for 10 min before returning to the initial conditions. MS data was acquired by means of information-dependent acquisition. MS scans were performed at 30,000 FWHM resolving power. The mass range of *m/z* 350–1250 was scanned over 250 ms and the highest 20 peaks were fragmented. A minimum accumulation time of 100 ms and total cycle time of 2.3 s was used for MS/MS. Also, the dynamic exclusion was set at 18 s.

### Data analysis

As previously described^28^, identification of proteins was performed using ProteinPilot™ 4.5 software (AB SCIEX) and Paragon™ algorithm (4.5.0.0.1654)^28^. Data were searched against a publicly available human UniProt database containing 20,210 protein entries (database: uniprot_sprot_20140122). The user-defined search parameters included: enzyme, trypsin; allowance of up to two missed cleavages; variable modification, methionine oxidation; fixed modification, carbamidomethyl cysteine; peptide mass tolerance, ± 20 ppm; fragment mass tolerance, 0.1 Da. Proteins were grouped using the ProGroup algorithm (AB SCIEX) to minimise redundancy. The false discovery rate (FDR) was estimated through an automatic decoy database search and was less than 1.0% for this study. To minimise false-positive data, a strict cutoff with an unused score ≥ 1.3 was applied. Proteins identified by two or more unique peptides with a 95% confidence or by one unique peptide with a 99% confidence were considered to be reliable^32, 33^. Redundant proteins and peptides as well as proteins identified by reverse sequence were discarded. To avoid apparent misidentifications resulting from protein name discrepancies, the gene names and UniProt accessions were manually examined^34^. Also, keratins were ignored and excluded in the final identification. All proteins and their matching unique peptides are listed in Supplementary Tables S1 and S2. The MS/MS spectra of the single unique peptide-based protein identifications are shown in the Supplementary Data. All raw and metadata of the proteome have been deposited to the iProx submission system (http://www.iprox.org) following the data-sharing policy of the ProteomeXchange consortium^35^. The data can be accessed from iProx with the identifier IPX00085101.

### Bioinformatics analysis

The p*I*/MW tool (http://web.expasy.org) was employed to calculate the theoretical isoelectric points (p*I*s) and molecular weights (MWs) of the identified proteins. Moreover, the protein interaction networks were mapped, and the corresponding physical and functional interactions were qualified using the web tool, Search Tool for the Retrieval of Interacting Genes/Proteins (STRING)^36^. The medium confidence (STRING score = 0.4) and seven linkage criteria were used for the network analysis^37^. A Cytoscape-Plugin Molecular Complex Detection (MCODE) analysis was further performed to identify highly connected regions in the generated network^38–40^.

## Results and Discussion

### Development of the TSP method for serum albuminome preparation

In albuminome research, chemical ethanol precipitation is commonly used for preparing an HSA-enriched fraction of human serum^10, 13, 19^. The remaining ethanol-soluble supernatant fraction obtained by direct ethanol precipitation is contaminated with IgG (Supplementary Fig. S1). Thus, effective enrichment of HSA using a conventional technique will depend on removal of IgG, and this issue must be addressed prior to using ethanol precipitation as the HSA-enrichment approach.

Previously, the protein G affinity column was commonly employed for IgG removal. In view of the aforementioned issue, herein we attempted to utilize the relatively inexpensive PEG. In our previous study, PEG precipitation was integrated into immunoaffinity depletion to enhance the detection of low-abundance human plasma proteins^41^. Unlike the majority of other agents, PEG precipitates protein components from natural mixtures by an exclusion mechanism^42^. Because the PEG precipitation procedure was performed at low temperature, the component types in the obtained fraction generally represent native protein complexes^43^. Because of these characteristics, PEG precipitation as a classical method was newly applied to remove IgG for albuminome preparation. PEG is able to effectively prevent the dissociation of the albuminome. Additionally, it is soluble in aqueous ethanol and thus probably compatible with downstream ethanol precipitation.

**Figure 1:**
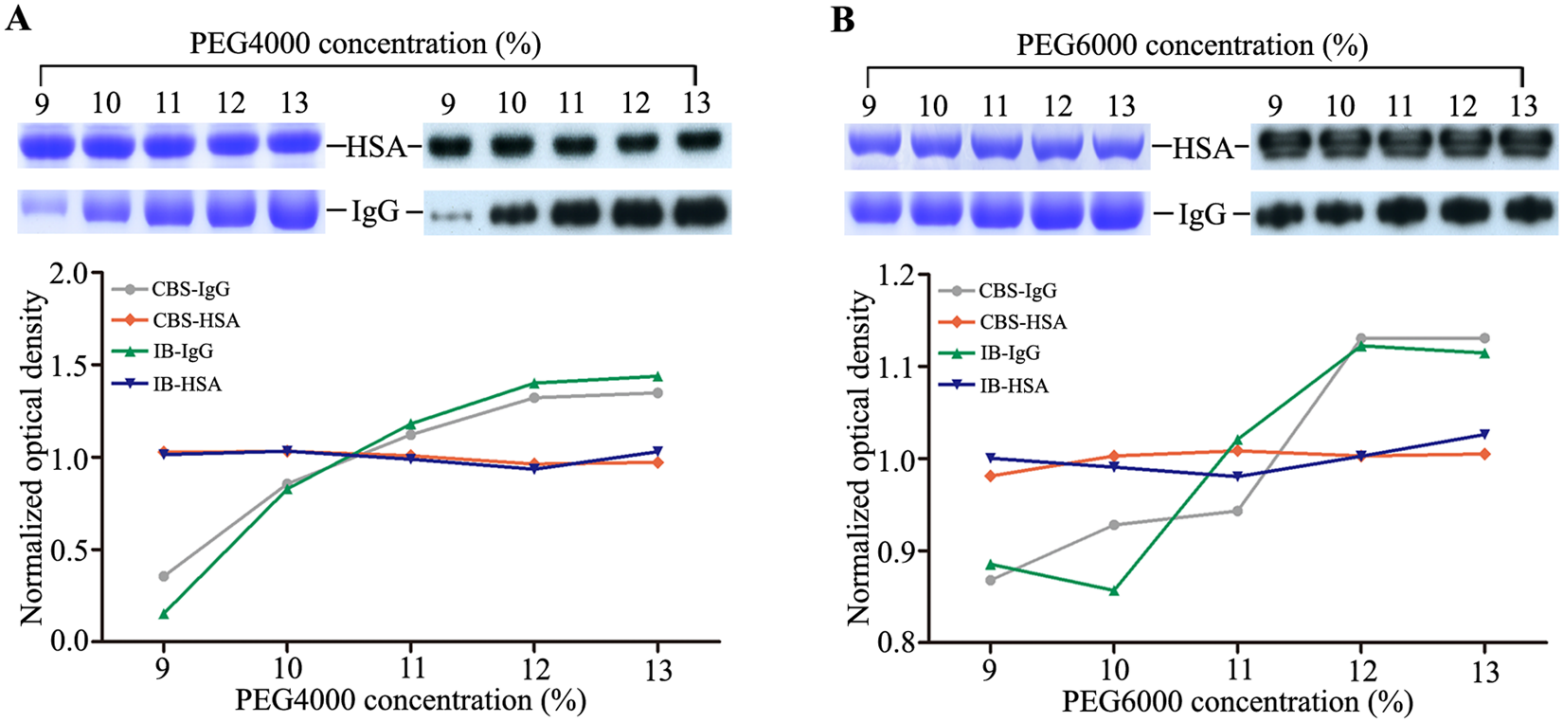
IgG removal of the uncrosslinked serum by various concentrations of PEG4000 (**A**) and PEG6000 (**B**). The resulting precipitate was analysed by SDS-PAGE with CBS, and immunoblotting with anti-IgG and anti-HSA antibodies. CBS-IgG, Coomassie blue stained IgG band; CBS-HSA, Coomassie blue stained HSA band; IB-IgG, immunoblotted IgG band; IB-HSA, immunoblotted HSA band.

To determine the optimal PEG concentration required for effective removal of IgG, the resulting precipitates at varying PEG concentrations (9–13%) were analysed by SDS-PAGE with Coomassie blue staining (CBS) and immunoblotting with an anti-IgG antibody (Fig. 1A and B). In this experiment, the two common protein precipitating agents, PEG4000 and PEG6000, were used simultaneously. From both the SDS-PAGE and immunoblotting data, we observed that PEG4000 and PEG6000 were equally effective at depleting IgG as the concentration of PEG increased to 12%. Moreover, immunoblotting with an anti-HSA antibody revealed that a solution containing PEG at a concentration of 9–13% did not result in any precipitation of HSA, suggesting that PEG precipitation will not disrupt the albuminome.

**Figure 2:**
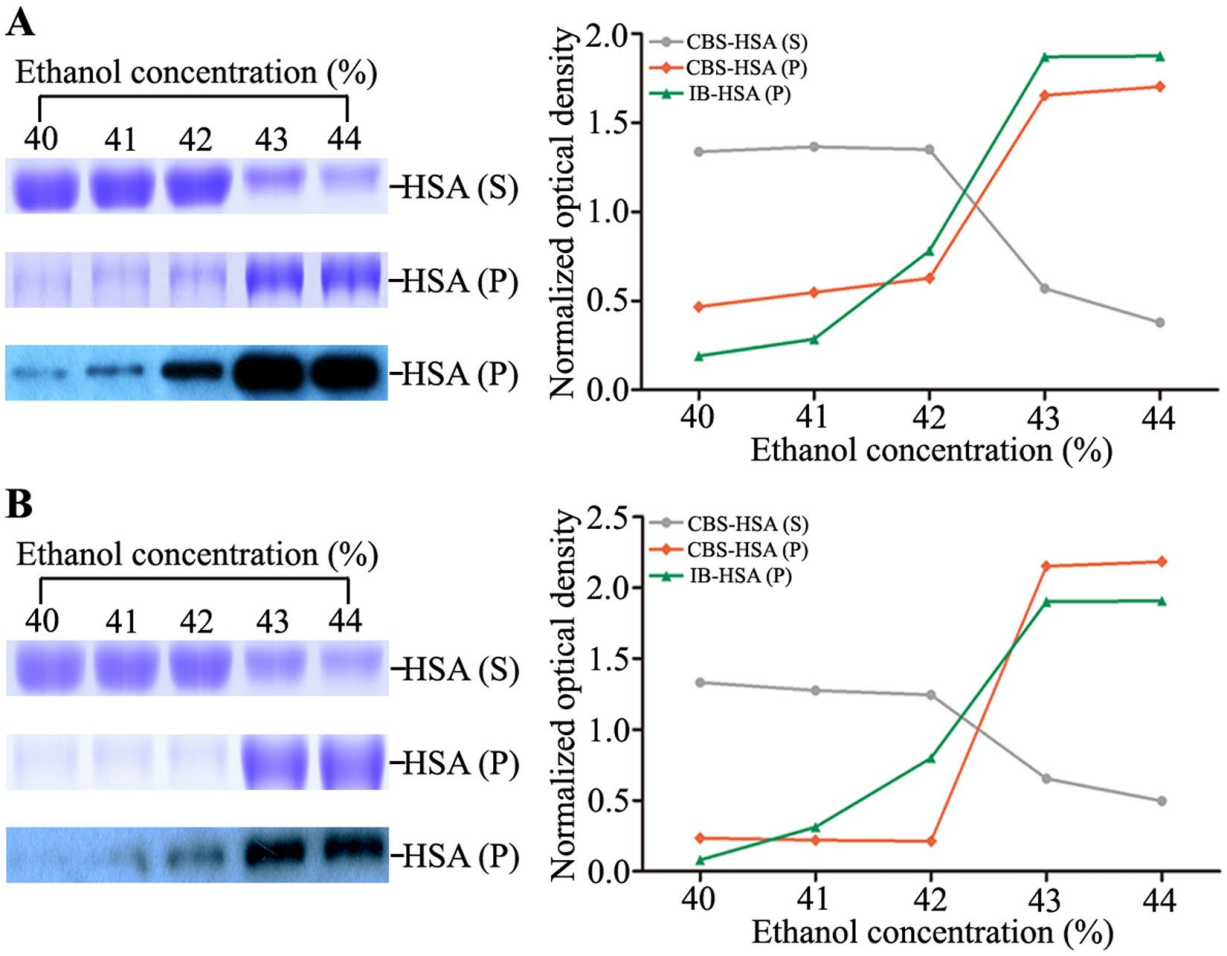
HSA enrichment of the uncrosslinked sample by various concentrations of ethanol after PEG4000 (**A**) and PEG6000 (**B**) precipitation. The supernatants and precipitates were analysed by SDS-PAGE with CBS and immunoblotted with the anti-HSA antibody. CBS-HSA (**S**), Coomassie blue stained HSA band of the supernatant; CBS-HSA (**P**), Coomassie blue stained HSA band of the precipitate; IB-HSA (**P**), immunoblotted HSA band of the precipitate.

After 12% PEG precipitation, ethanol precipitation was performed to prepare the serum albuminome as described previously^19^. The above-prepared supernatants were further precipitated by the addition of various concentrations of ethanol, and the resulting precipitates were then subjected to SDS-PAGE with CBS and immunoblotting with the anti-HSA antibody (Fig. 2A and B). We observed that a large amount of HSA suddenly precipitated when the ethanol concentration reached 43%, which was in accordance with previous studies^13, 19^. In contrast, this significant difference was simultaneously detected in the ethanol-soluble supernatants. These results also suggested that the introduction of PEG into the preparation of the HSA-enriched fraction did not seem to have any superimposed effect on downstream ethanol precipitation. Thus, a TSP procedure consisting of an initial 12% PEG precipitation step followed by the addition of 42% ethanol is useful for isolating the serum albuminome. Moreover, the use of PEG4000 or PEG6000 resulted in no difference with respect to the serum albuminome isolation. Furthermore, the utility of the TSP protocol was demonstrated by SDS-PAGE analysis of the serum albuminomes obtained from three healthy donors. As expected, the HSA band was found to dominate the ethanol-soluble supernatant fractions (Supplementary Fig. S2). Using the HSA band as reference, we found that 69.6 ± 11.4% (mean ± SD, similarly hereinafter) of HSA with PEG4000 and 71.3 ± 6.8% with PEG6000 were enriched from the whole serum.

### Integrated use of FC and TSP for fixed preparation of the serum albuminome

In the TSP protocol, the serum sample was subjected to nonphysiological conditions due to addition of the chemical agents. Structural integrity of the obtained albuminome might be affected to some extent. To maintain the integrity, FC was integrated into the designed TSP procedure. Formaldehyde is a zero-length crosslinker that rapidly inactivates proteins to ensure the stability of complexes^44^. Only closely related proteins can be crosslinked owing to the small size of formaldehyde^27^. The crosslinks are reversible, enabling enzymatic digestion and MS detection of the proteins within a complex sample^44^. Formaldehyde is also believed to allow very quick crosslinking and essentially freeze transient interactions^26^. Generally, the formaldehyde concentration and reaction time can be tuned as two complementary parameters to achieve efficient crosslinking.

In the current study, a 5-s fixation period was initially tested to achieve rapid crosslinking. The lipid-depleted serum was added directly to different concentrations of formaldehyde. When a 5-s quick cross-linking period was used, we observed a significantly different appearance by using formaldehyde at a concentration of ≥ 5% compared with the control (Supplementary Fig. S3A). When 10% and higher formaldehyde concentrations were used, the obtained patterns were similar, suggesting that 10% was sufficient to fix the interactions in the serum (Supplementary Fig. S3B). It was also observed that the patterns obtained using longer reaction times in 10% formaldehyde had no apparent differences, indicating that the 5-s incubation time was sufficient for crosslinking. For the following experiments, a 5-s crosslinking period in 10% formaldehyde was chosen.

**Figure 3:**
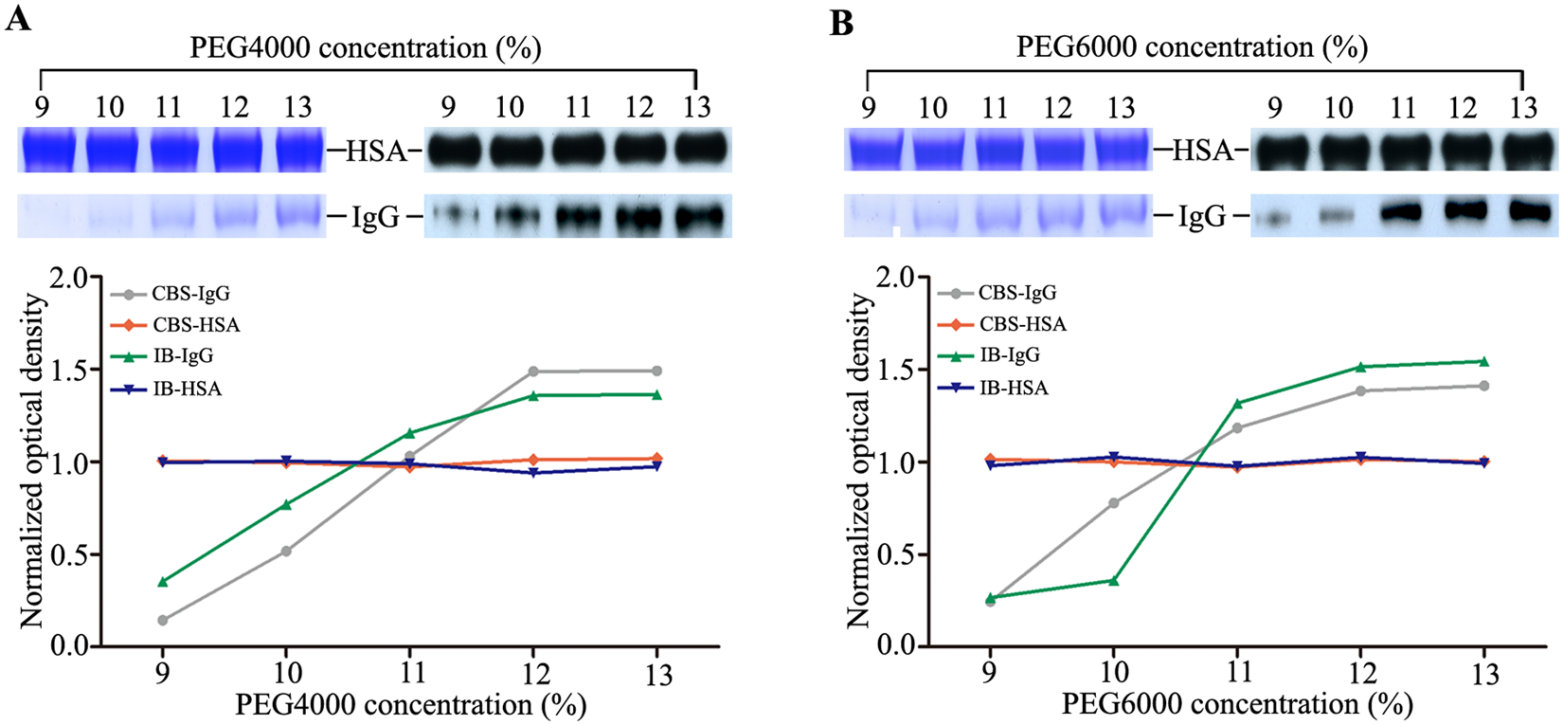
IgG removal of the crosslinked serum by various concentrations of PEG4000 (**A**) and PEG4000 (**B**). The resulting precipitates were analysed by SDS-PAGE with CBS and immunoblotted with anti-IgG and anti-HSA antibodies. The abbreviations used are the same as those used in Fig. 1.

After FC-based quick fixation, the crosslinked serum was used to prepare the albuminome through the TSP procedure. Likewise, PEG precipitation was a necessary step to eliminate IgG contamination before using ethanol precipitation (Supplementary Fig. S1). Accordingly, the crosslinked sample was incubated with different PEG concentrations for IgG removal. The PEG-precipitated pellets were analysed by SDS-PAGE and immunoblotted to determine the optimal concentration of PEG required (Fig. 3A and B). IgG had fully precipitated when either PEG4000 or PEG6000 was used at 12%, which is a similar result to the aforementioned uncrosslinked samples. We also observed that PEG (9–13%) did not induce any precipitation of HSA. As a note, only detection of the IgG heavy chain with the corresponding antibody was used. Nevertheless, SDS-PAGE analysis of the PEG-precipitated pellet fractions showed that the IgG heavy and light chains were simultaneously precipitated as potential complexes in either uncrosslinked or crosslinked samples (Supplementary Fig. S4).

**Figure 4:**
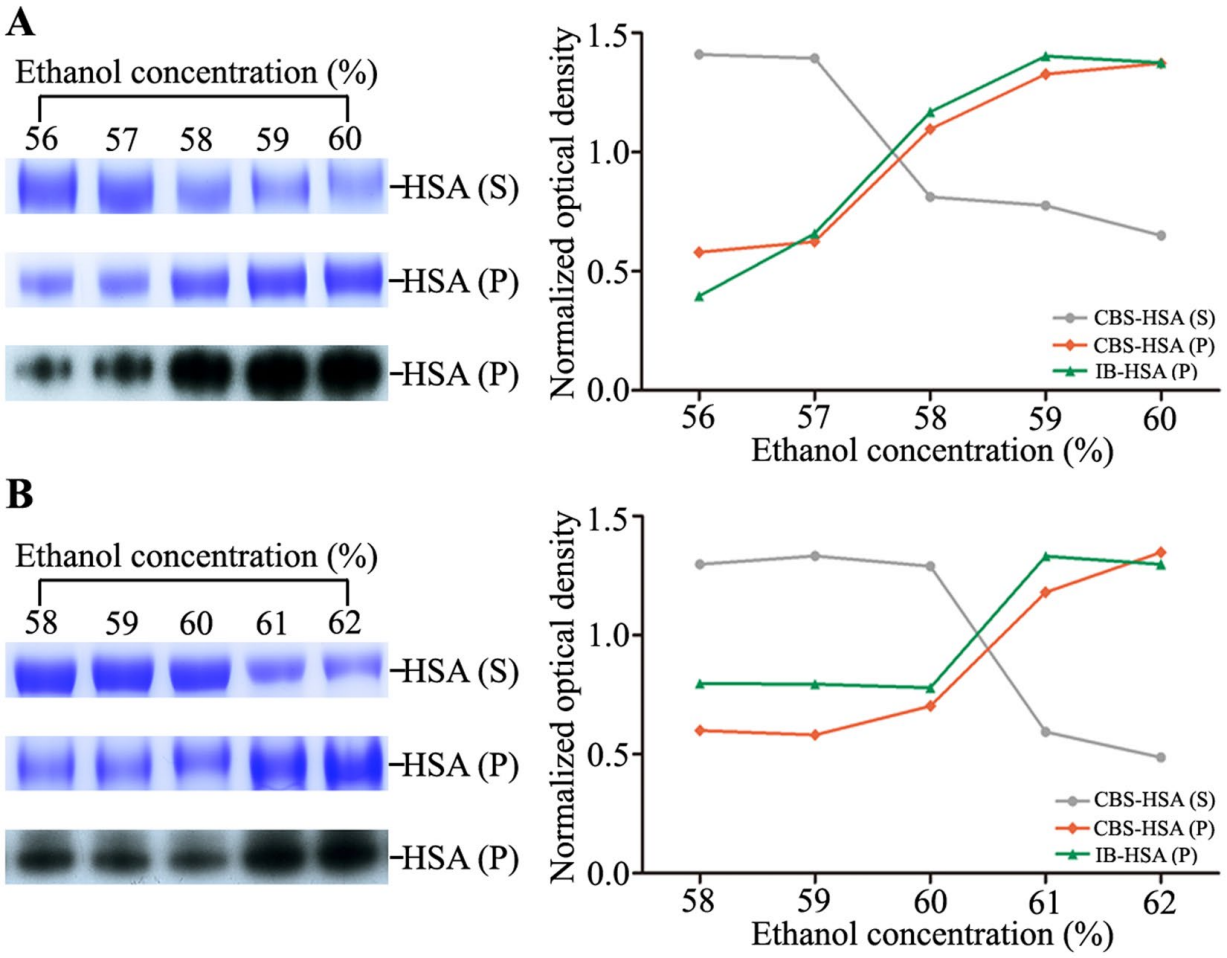
HSA enrichment of the crosslinked sample by various concentrations of ethanol after PEG4000 (**A**) and PEG6000 (**B**) precipitation. The supernatant and precipitate were analysed by SDS-PAGE with CBS and immunoblotted with the anti-HSA antibody. The abbreviations used are the same as those used in Fig. 2.

**Figure 5:**
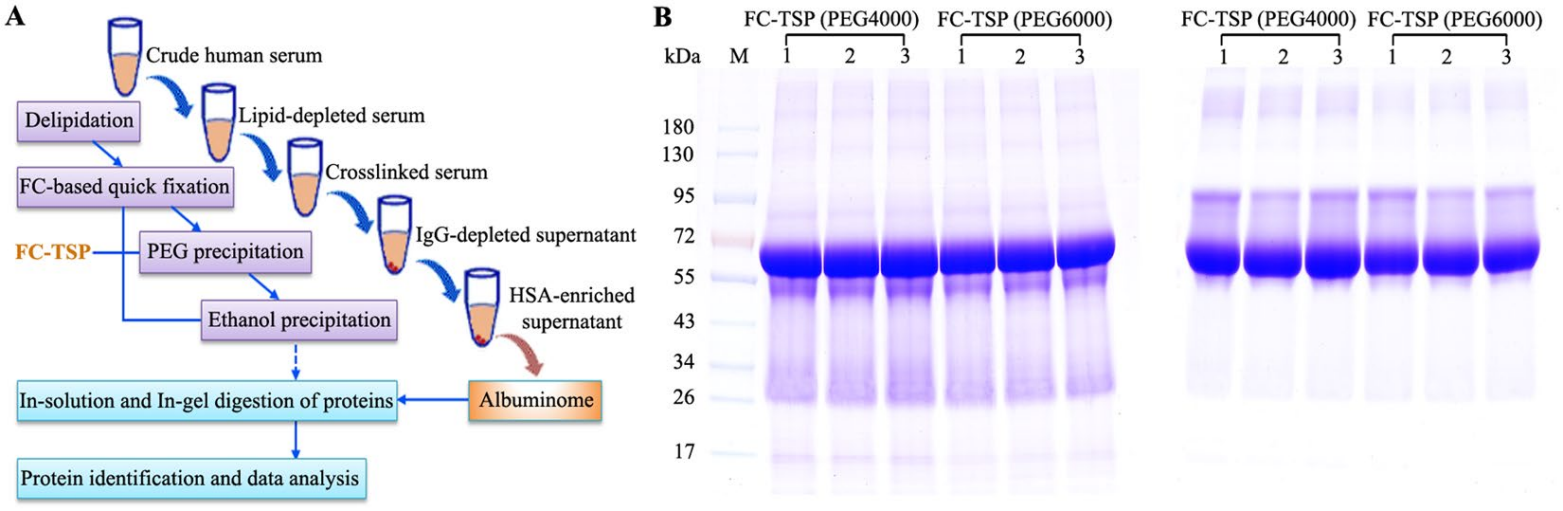
FC-TSP approach for the analysis of the human serum albuminome. (**A**) Flow chart of the approach. After delipidation by centrifugation, the serum was quickly fixed by the FC procedure. The TSP protocol that includes PEG precipitation followed by ethanol precipitation was then used to prepare the albuminome. The obtained fraction was subjected to trypsin digest and the resultant peptides were analysed by LC-MS/MS. (**B**) SDS-PAGE analysis of serum albuminomes from three healthy individuals using this approach. The crosslinked samples were respectively heated at 95 °C (left) and 65 °C (right). Lane 1, a 55-year-old woman; lane 2, a 35-year-old woman; lane 3, a 54-year-old man; M, MW marker.

After 12% PEG precipitation, we continued to probe into the required ethanol concentration for albuminome preparation of the crosslinked samples (Fig. 4A and B). In contrast to the uncrosslinked samples, HSA from the PEG4000-treated crosslinked samples was not precipitated until the ethanol concentration reached 58%. Similarly, in the PEG6000-treated crosslinked samples, a large amount of HSA precipitant was not observed until the ethanol concentration reached 61%. In contrast, these observed significant differences were detected simultaneously in the corresponding ethanol-soluble supernatants. The results demonstrated that for the crosslinked sample, the serum albuminome can be produced by simply using ethanol concentrations of 57% and 60% after precipitation with 12% PEG4000 and PEG6000, respectively. Accordingly, development of this FC-TSP method provides a notable integration of features amendable to the analysis of native HSA-binding proteins (Fig. 5A). Further, SDS-PAGE analysis of the serum albuminomes obtained from three healthy individuals by the FC-TSP approach demonstrated the utility of the method with good reproducibility (Fig. 5B). Of note, protein bands from the samples treated at 65 °C were mainly distributed in the HSA and its above portion, suggesting that the formaldehyde crosslinks were conserved. Similar to the TSP method, 70.6 ± 7.3% of HSA with PEG4000 and 72.4 ± 2.4% with PEG6000 were enriched from the whole serum. Together, these results indicate that this new approach can be used as a powerful tool to process clinical samples for albuminome analysis.

### FC-TSP combined with MS-based and network-based analyses for characterisation of human serum albuminome

**Figure 6:**
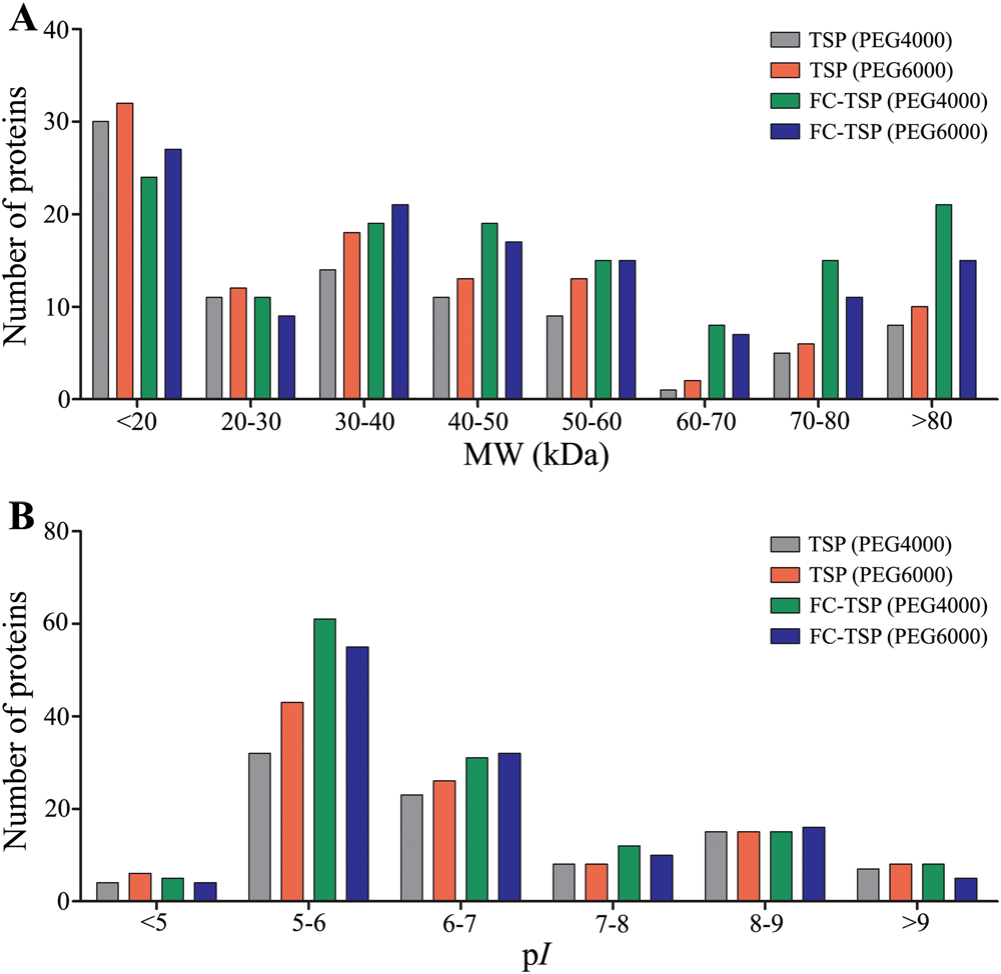
Comparison of the distributions of the serum albuminome proteins identified using the TSP and FC-TSP methods as a function of MW (**A**) and p*I* (**B**). Detailed protein identifications are listed in Supplementary Table S1.

In the present study, utility of the FC procedure for identification of serum albuminome was initially assessed by solution-based LC-MS/MS analysis. In contrast to the non-crosslinking TSP method, introduction of the FC procedure resulted in the identification of more proteins, demonstrating that crosslinking can potentially capture real-time weak and transient interactions. To further investigate the effect of FC on the physicochemical characterisation of the human serum albuminome, MW and p*I* values of the identified proteins from the TSP and FC-TSP methods were compared as shown in Fig. 6. Compared with the TSP method, more proteins in the MW range of more than 40 kDa and p*I* range of 5–8 were identified using the FC-TSP method, suggesting that the TSP method might lead to a loss of relatively high MW proteins. We also observed that there was no significant difference between PEG4000 and PEG6000.

**Figure 7:**
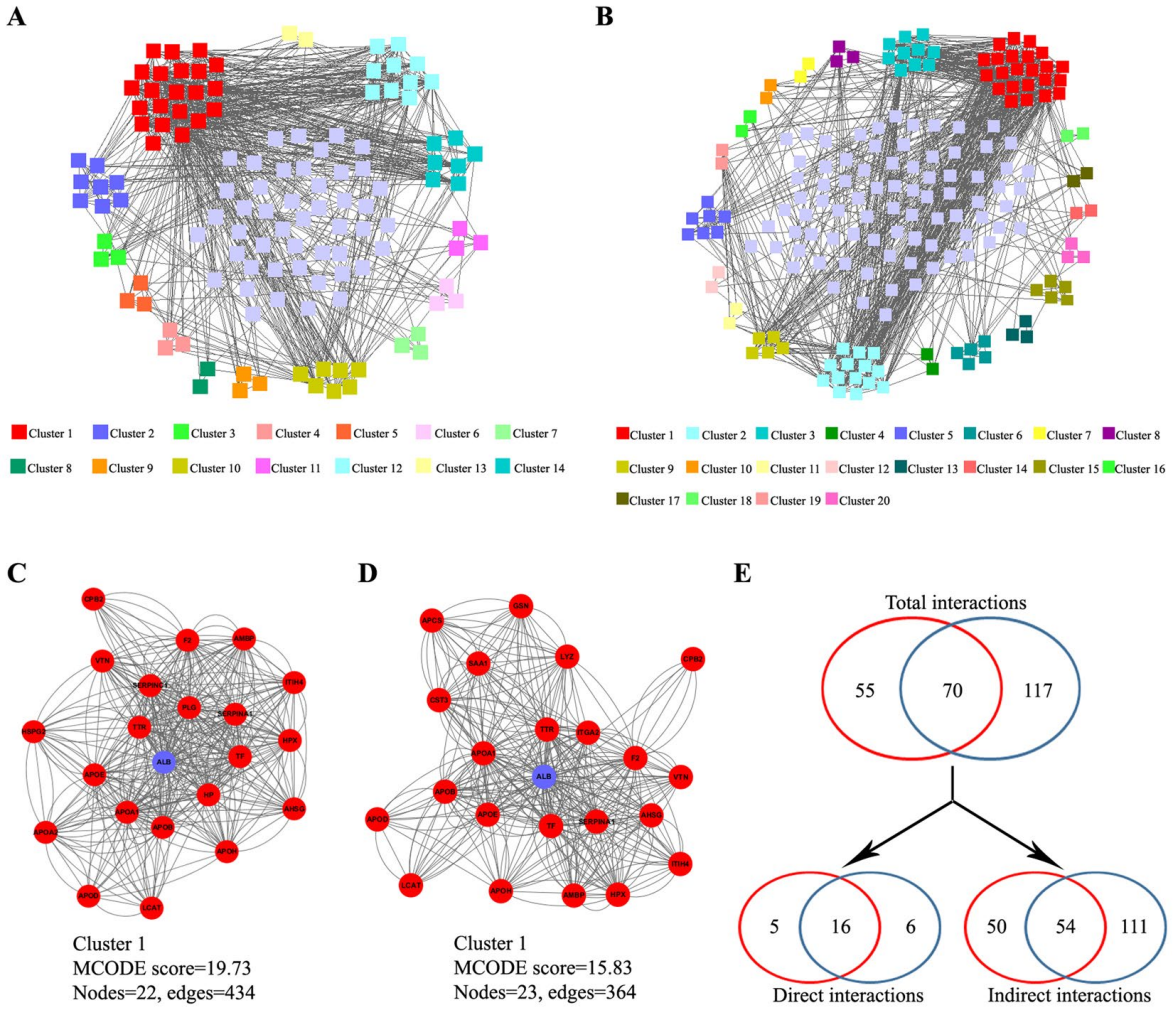
Comparison of HSA-interacting proteins identified in this study combined with those identified in five previous studies. Two complete interaction networks from our (**A**) and the five other combined (**B**) proteomics data were obtained from the STRING database. (**C**) and (**D**) showing the corresponding HSA-containing clusters obtained with MCODE for this study and the five other studies, respectively. (**E**) Venn diagram comparing the number of HSA-related proteins identified by our (red circle) and the five previous (blue circle) studies with some direct and indirect interactions.

To comprehensively profile human serum albuminome, the HSA-enriched fraction obtained by the FC-TSP method was simultaneously analysed by solution-based and gel-based approaches with highly sensitive MS^45, 46^. Protein identification results were merged and listed in Supplementary Table S3. A total of 171 proteins excluding HSA were identified based on two or more unique peptides with 95% confidence or one unique peptide with 99% confidence, which including some high and low abundance proteins^32, 33^. To generate the HSA protein interaction network at a systems-level, all identified proteins were imported into a STRING database of physical and functional interactions^36^. The interaction network was then built using Cytoscape as shown in Fig. 7 ^40^. A total of 125 (73%) of HSA-interacting proteins were mapped to the interaction network either directly or indirectly^10^. These proteins were considered to be intrinsically associated with HSA and some were likely albuminome protein candidates. In the interaction map, the connected proteins tended to form several highly-linked clusters (i.e., sub-networks)^38^. Further, a MCODE analysis was executed to identify 14 highly-connected clusters (Fig. 7A; Supplementary Table S4). In general, these formed clusters may represent multiprotein complexes that actually carry out cellular functions and processes^47^. As shown in Fig. 7C, HSA formed a complex with 21 proteins (cluster 1). In fact, the albuminome refers to the proteome of HSA-associated partners and simultaneously includes direct and indirect binders. It is therefore tempting to speculate that the 21 proteins in cluster 1 could be direct HSA-binders. The other 104 proteins present in the network likely resulted from an indirect association with HSA, and yielded 13 putative protein complexes. In the network, interactions between all these complexes may indeed importantly contribute to coordination and cooperation of their functions. Taken together, the results of the present study indicate that our FC-TSP approach is an effective tool that can be used to characterize the albuminome. Subsequent network analysis might be needed to further improve our knowledge of the human albumin interactome.

### Discovery of new potential albuminome proteins by comparison with previous albuminome studies

First, the 171 proteins identified using the FC-TSP method were initially compared with the data of five previous major albuminome studies (Supplementary Fig. S5 and Table S2)^10, 11, 19, 20, 48^. In these studies, the employed strategies were generally based on affinity purification or chemical-based methods. Comparison of our results with those of the previous study using the protein G-ethanol method revealed that up to 92% of the proteins from the previous study^19^ were identified in our study and 125 proteins were identified only by our method. From an overall comparison, we observed that 99 (58%) proteins from our results were also identified in all previous studies. Our data were partially complementary with previous reports, suggesting that our new method can be used as an alternative strategy for identifying proteins in the albuminome. In the six studies, the variation in the number and types of identified proteins may be attributed to the different experimental conditions (*i*.*e*., serum source, enrichment methods, MS instrumentation, search engines and parameters).

In the present study, the proteomics data from the five previous reports were further combined. A total of 276 proteins were obtained and then used for the network construction in an effort to discover some proteins intrinsically associated with HSA. As shown in Fig. 7B and Supplementary Table S4, 187 (68%) potential albuminome components were found with STRING and the corresponding 20 putative clusters were further revealed with MCODE. Similarly, these identified albuminome proteins contained 22 direct and 165 indirect binders (Fig. 7D). Subsequently, a side-by-side comparison of albuminome proteins obtained from our study and previous studies was carried out as shown in Fig. 7E. A total of 55 proteins were exclusively identified in our study, which included five direct and 50 indirect binders. The proteins identified only by our FC-TSP strategy may be potential new albuminome components. Further studies are required to verify candidates by use of protein-protein interaction techniques.

Human serum albuminome is a known valuable source for the identification of clinically informative biomarkers. Proteins that bind to HSA are predicted to change as a result of disease states^7, 8^. These HSA-binding proteins as indicators of a pathophysiological state may be present at lower concentrations in circulating blood. We observed that the albuminomes from the six studies included 28 known low-abundance proteins (LAPs) with concentrations below 100 ng/mL (based on published data) (Supplementary Table S5)^49–53^. Twelve of these LAPs were identified exclusively in our study, and included candidate cancer biomarkers, such as CDH5, VCAM1, and IGFBP3^50, 54–57^. IGFBP3 could also be applied to assessment of preoperative depression risk of high-grade glioma patients^58^. The capture of albuminome LAPs should aid in the discovery of potential candidate biomarkers, and the FC-TSP approach should be suitable for real-time capturing of LAPs from the albuminome that are of clinical interest.

## Conclusion

In this study, we first developed a modified TSP method to replace the conventional protein G-ethanol method for effective preparation of serum albuminome. To capture weak and transient interactions in real time, we further optimised the well-established protocol by combining FC and TSP. We have shown here that the newly developed FC-TSP approach can be used for comprehensive profiling of human serum albuminome. This method is simple, rapid, inexpensive and easy to use, and appropriate for albuminome preparation of multiple clinical samples. We anticipate that the FC-TSP-MS strategy will have widespread future applications for albuminome-based biomarker discovery.

## Electronic supplementary material

Supplementary Information

Supplementary Table S1

Supplementary Table S2

Supplementary Table S3

Supplementary Table S4

Supplementary Table S5

Supplementary Data
