# Supplementary material for: Proteomic and network analysis of human serum albuminome by integrated use of quick crosslinking and two-step precipitation
Source: Sci Rep. 2017 Aug 29;7:9856. doi: 10.1038/s41598-017-09563-w (PMC5575314; doi:10.1038/s41598-017-09563-w)
Supplement: Supplementary file 1 — Supplementary Information [file 41598_2017_9563_MOESM1_ESM.doc]

**Proteomic and network analysis of human serum** **albuminome by integrated use of** **quick crosslinking and** **two-step precipitation**

Zhao Liu^1,2,3§^, Shuiming Li^4§^, Haiyang Wang^1,2,3§^, Min Tang^2,3^, Mi Zhou^2,3^, Jia Yu^2,3^, Shunjie Bai^2,3^, Pengfei Li^2,3^, Jian Zhou^2,3*^, Peng Xie^1,2,3,5*^

^1^Department of Neurology, the First Affiliated Hospital of Chongqing Medical University, Chongqing 400016, China

^2^Institute of Neuroscience and the Collaborative Innovation Center for Brain Science, Chongqing Medical University, Chongqing 400016, China

^3^Chongqing Key Laboratory of Neurobiology, Chongqing 400016, China

^4^Shenzhen Key Laboratory of Microbiology and Gene Engineering, Shenzhen University, Shenzhen 518060, China

^5^South Australian Health and Medical Research Institute (SAHMRI), North Terrace, Adelaide, SA 5000, Australia

^§^These authors contributed equally to this work.

^*^To whom correspondence should be addressed:

Institute of Neuroscience and the Collaborative Innovation Center for Brain Science, Chongqing Medical University, 1 Yixueyuan Road, Yuzhong District, Chongqing 400016, China (J. Zhou); Department of Neurology, the First Afﬁliated Hospital of Chongqing Medical University, 1 Yixueyuan Road, Yuzhong District, Chongqing 400016, China (P. Xie). Tel: +86-23-68485490, Fax: +86-23-68485111.

E-mail addresses: zhoujian@cqmu.edu.cn (J. Zhou), xiepeng@cqmu.edu.cn (P. Xie).

**Supplementary** **Information**

**Supplementary Figure S1.** SDS-PAGE analysis of the supernatant fractions prepared by direct ethanol precipitation. The ethanol-soluble supernatant fractions from either uncrosslinked or crosslinked samples are contaminated with IgG heavy and light chains. M, MW marker.

**Supplementary Figure S2.** SDS-PAGE analysis of serum albuminomes from three healthy individuals by the TSP method. Lane 1, a 55-year-old woman; lane 2, a 35-year-old woman; lane 3, a 54-year-old man; M, MW marker.

**Supplementary Figure S3.** FC-based fixation of serum samples. The serum was incubated with different concentrations of formaldehyde for 5 s (A) or with 10% formaldehyde for different periods (B) and then analysed by SDS-PAGE.

**Supplementary Figure S4.** SDS-PAGE analysis of the resulting precipitates from the human serum sample following the addition of 12% PEG4000 and PEG6000. The image shows that the IgG heavy and light chains from either uncrosslinked or crosslinked samples were simultaneously precipitated. M, MW marker.

**Supplementary Figure S5.** Comparison of the serum proteins identified in this study with those identified in five previous studies of the albuminome. Total proteins identified from the respective studies are shown as Venn diagrams. The protein descriptions are shown in Supplementary Table S3.

**Supplementary Table S1.** List of the proteins identified from the HSA-enriched fraction obtained using the TSP and FC-TSP methods.

**Supplementary Table S2.** List of the unique peptides corresponding to the identified proteins as shown in Supplementary Table S1.

**Supplementary Table S3.** Complete list of all proteins identified from the six studies of the albuminome including our study.

**Supplementary Table S4.** Clusters of the STRING interaction network of proteomics data from our and five previous studies generated using MCODE analysis.

**Supplementary Table S5.** Comparison of the known LAPs with reported concentrations below 100 ng/mL (based on previously published data) identified as HSA-interacting proteins from our and five previous studies.

**Supplementary Data.** The MS/MS spectra of single unique peptide-based protein identifications in this study.


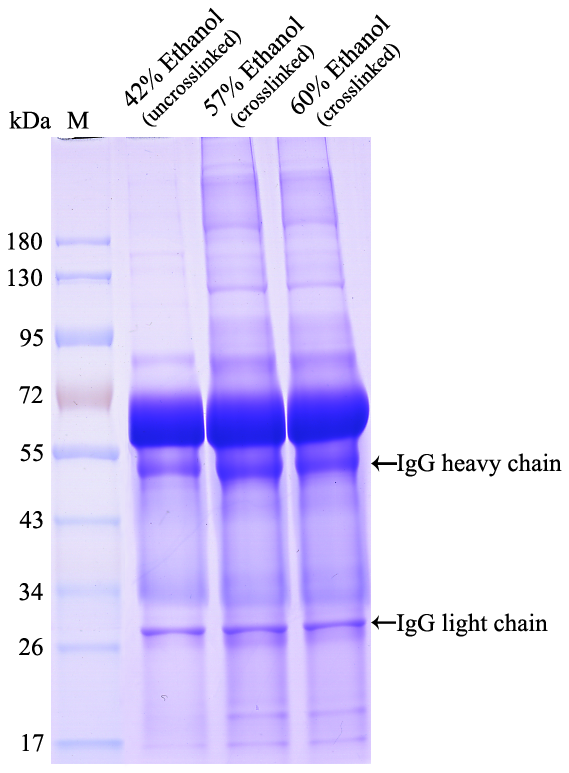


**Supplementary Figure S1**

**
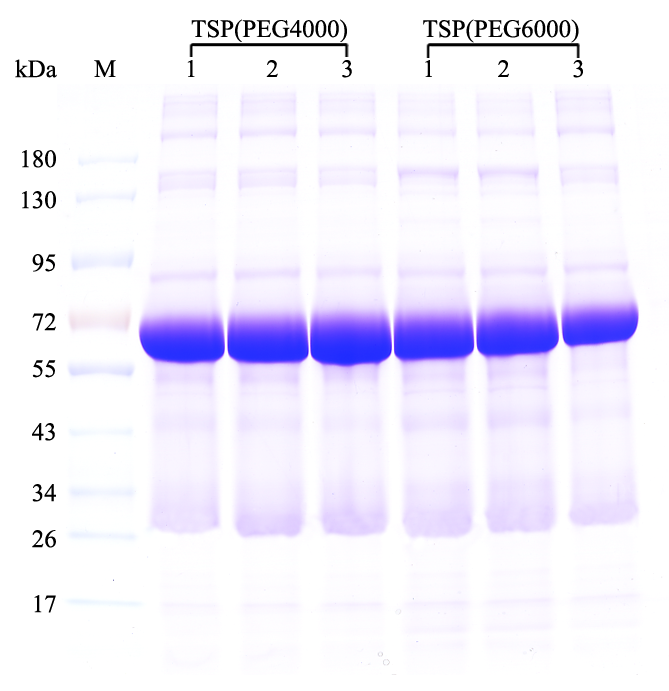
**

**Supplementary Figure S2**

**
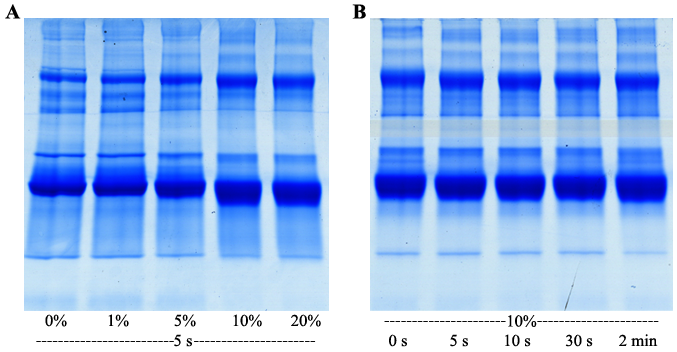
**

**Supplementary Figure S3**

**
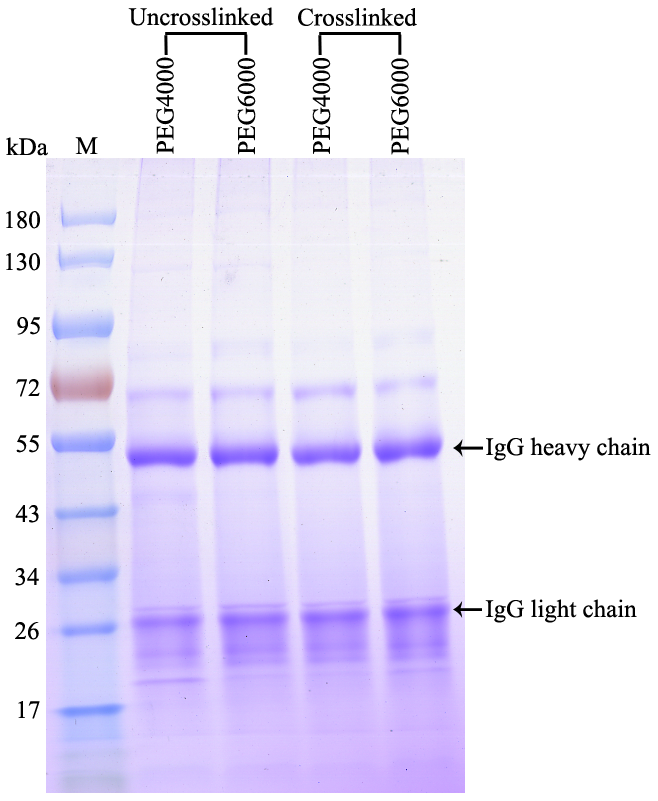
**

**Supplementary Figure S4**

**
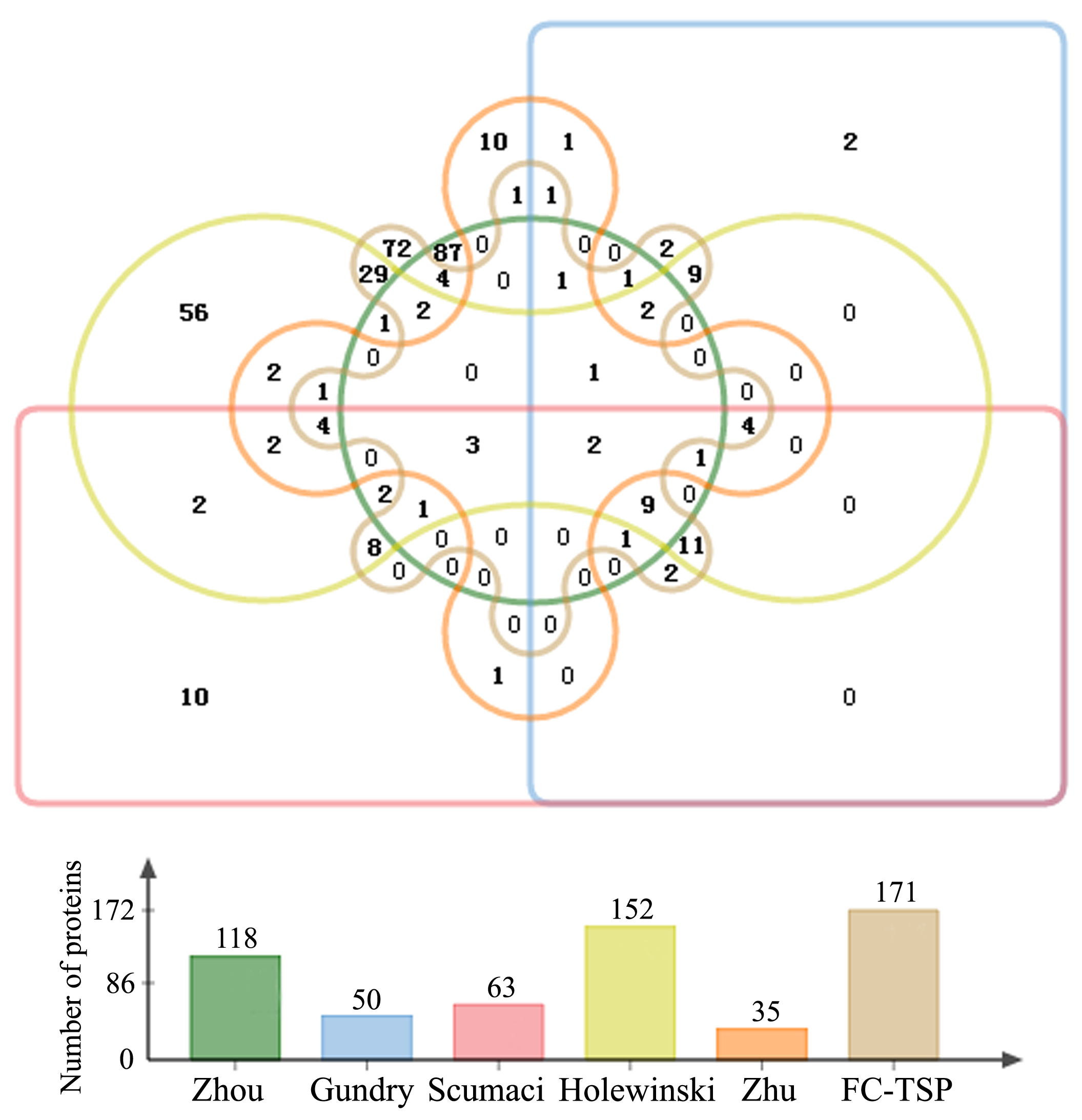
**

**Supplementary Figure S5**
