# Supplementary material for: Proteomic and network analysis of human serum albuminome by integrated use of quick crosslinking and two-step precipitation
Source: Sci Rep. 2017 Aug 29;7:9856. doi: 10.1038/s41598-017-09563-w (PMC5575314; doi:10.1038/s41598-017-09563-w)
Supplement: Supplementary file 7 — Supplementary Data [file 41598_2017_9563_MOESM7_ESM.doc]

TSP (PEG4000)-solution-rep 1

64

| Contrib | Conf | Sequence | Modifications | Cleavages | ∆Mass | Prec MW | z | Sc | Spectrum | Type |
| --- | --- | --- | --- | --- | --- | --- | --- | --- | --- | --- |
| 2 | 99 | ADGLAVIGVLMK |  |  | -0.0003 | 1185.679 | 2 | 13 | 1.1.1.4176.7 | Winner |

65

| Contrib | Conf | Sequence | Modifications | Cleavages | ∆Mass | Prec MW | z | Sc | Spectrum | Type |
| --- | --- | --- | --- | --- | --- | --- | --- | --- | --- | --- |
| 2 | 99 | GLGELQELYLK |  |  | -0.001 | 1261.691 | 2 | 17 | 1.1.1.3868.5 | Winner |

66

| Contrib | Conf | Sequence | Modifications | Cleavages | ∆Mass | Prec MW | z | Sc | Spectrum | Type |
| --- | --- | --- | --- | --- | --- | --- | --- | --- | --- | --- |
| 2 | 99 | TPEVTCVVVDVSHEDPEVQFK | Carbamidomethyl(C)@6 |  | 0.0055 | 2413.153 | 3 | 18 | 1.1.1.3836.38 | Winner |

67

| Contrib | Conf | Sequence | Modifications | Cleavages | ∆Mass | Prec MW | z | Sc | Spectrum | Type |
| --- | --- | --- | --- | --- | --- | --- | --- | --- | --- | --- |
| 2 | 99 | DASGATFTWTPSSGK |  |  | -0.0003 | 1511.689 | 2 | 19 | 1.1.1.3446.24 | Winner |

68

| Contrib | Conf | Sequence | Modifications | Cleavages | ∆Mass | Prec MW | z | Sc | Spectrum | Type |
| --- | --- | --- | --- | --- | --- | --- | --- | --- | --- | --- |
| 2 | 99 | AAPSVTLFPPSSEELQANK |  |  | -0.0008 | 1985.01 | 2 | 19 | 1.1.1.3684.8 | Winner |

69

| Contrib | Conf | Sequence | Modifications | Cleavages | ∆Mass | Prec MW | z | Sc | Spectrum | Type |
| --- | --- | --- | --- | --- | --- | --- | --- | --- | --- | --- |
| 2 | 99 | EPFLSCCQFAESLR | Carbamidomethyl(C)@6; Carbamidomethyl(C)@7 |  | -0.0001 | 1742.776 | 2 | 16 | 1.1.1.3850.16 | Winner |

71

| Contrib | Conf | Sequence | Modifications | Cleavages | ∆Mass | Prec MW | z | Sc | Spectrum | Type |
| --- | --- | --- | --- | --- | --- | --- | --- | --- | --- | --- |
| 2 | 99 | FLVGPDGIPIMR |  |  | 0.0025 | 1313.719 | 2 | 11 | 1.1.1.3950.10 | Winner |

72

| Contrib | Conf | Sequence | Modifications | Cleavages | ∆Mass | Prec MW | z | Sc | Spectrum | Type |
| --- | --- | --- | --- | --- | --- | --- | --- | --- | --- | --- |
| 2 | 99 | FPSLRAVPAYGR |  | missed R-A@5 | -0.0102 | 1332.72 | 2 | 8 | 1.1.1.4671.5 | Winner |

73

| Contrib | Conf | Sequence | Modifications | Cleavages | ∆Mass | Prec MW | z | Sc | Spectrum | Type |
| --- | --- | --- | --- | --- | --- | --- | --- | --- | --- | --- |
| 2 | 99 | FIIEGMEEAGSVALEELVEK |  |  | 0.0041 | 2192.096 | 3 | 13 | 1.1.1.4521.12 | Winner |

74

| Contrib | Conf | Sequence | Modifications | Cleavages | ∆Mass | Prec MW | z | Sc | Spectrum | Type |
| --- | --- | --- | --- | --- | --- | --- | --- | --- | --- | --- |
| 2 | 99 | QRPIPLSMIFK |  |  | 0.0096 | 1328.773 | 2 | 9 | 1.1.1.4415.3 | Winner |

75

| Contrib | Conf | Sequence | Modifications | Cleavages | ∆Mass | Prec MW | z | Sc | Spectrum | Type |
| --- | --- | --- | --- | --- | --- | --- | --- | --- | --- | --- |
| 2 | 99 | VGAHAGEYGAEALER |  |  | -0.002 | 1528.725 | 3 | 19 | 1.1.1.2970.8 | Winner |

76

| Contrib | Conf | Sequence | Modifications | Cleavages | ∆Mass | Prec MW | z | Sc | Spectrum | Type |
| --- | --- | --- | --- | --- | --- | --- | --- | --- | --- | --- |
| 2 | 99 | QVQLVQSGAEVK | Gln->pyro-Glu@N-term | cleaved S-Q@N-term | -0.0002 | 1267.677 | 2 | 19 | 1.1.1.3436.17 | Winner |

77

| Contrib | Conf | Sequence | Modifications | Cleavages | ∆Mass | Prec MW | z | Sc | Spectrum | Type |
| --- | --- | --- | --- | --- | --- | --- | --- | --- | --- | --- |
| 2 | 99 | EVQLVESGGGLVQPGGSLR |  |  | 0.0014 | 1880.997 | 2 | 27 | 1.1.1.3560.24 | Winner |

78

| Contrib | Conf | Sequence | Modifications | Cleavages | ∆Mass | Prec MW | z | Sc | Spectrum | Type |
| --- | --- | --- | --- | --- | --- | --- | --- | --- | --- | --- |
| 2 | 99 | EVQLLESGGGLVQPGGSLR |  | cleaved C-E@N-term | -0.005 | 1895.006 | 2 | 23 | 1.1.1.3725.27 | Winner |

79

| Contrib | Conf | Sequence | Modifications | Cleavages | ∆Mass | Prec MW | z | Sc | Spectrum | Type |
| --- | --- | --- | --- | --- | --- | --- | --- | --- | --- | --- |
| 2 | 99 | DIQMTQSPSSLSASVGDR |  | cleaved C-D@N-term | 0.0046 | 1877.883 | 2 | 19 | 1.1.1.3412.18 | Winner |

80

| Contrib | Conf | Sequence | Modifications | Cleavages | ∆Mass | Prec MW | z | Sc | Spectrum | Type |
| --- | --- | --- | --- | --- | --- | --- | --- | --- | --- | --- |
| 2 | 99 | VQPVSEILQLKTYLPTFETTI |  | missed K-T@11 | -0.0127 | 2419.312 | 3 | 7 | 1.1.1.4428.21 | Winner |

81

| Contrib | Conf | Sequence | Modifications | Cleavages | ∆Mass | Prec MW | z | Sc | Spectrum | Type |
| --- | --- | --- | --- | --- | --- | --- | --- | --- | --- | --- |
| 2 | 99 | LSNFLHAQQWKGASNYVAK |  | missed K-G@11 | -0.0005 | 2161.106 | 3 | 9 | 1.1.1.4383.8 | Winner |

82

| Contrib | Conf | Sequence | Modifications | Cleavages | ∆Mass | Prec MW | z | Sc | Spectrum | Type |
| --- | --- | --- | --- | --- | --- | --- | --- | --- | --- | --- |
| 2 | 99 | TLAVLLDNILQR | Deamidated(N)@8 |  | 0.0052 | 1368.803 | 2 | 11 | 1.1.1.4423.8 | Winner |

83

| Contrib | Conf | Sequence | Modifications | Cleavages | ∆Mass | Prec MW | z | Sc | Spectrum | Type |
| --- | --- | --- | --- | --- | --- | --- | --- | --- | --- | --- |
| 2 | 99 | SNFLNCYVSGFHPSDIEVDLLK | Carbamidomethyl(C)@6 |  | 0.0016 | 2553.223 | 3 | 21 | 1.1.1.4303.7 | Winner |

84

| Contrib | Conf | Sequence | Modifications | Cleavages | ∆Mass | Prec MW | z | Sc | Spectrum | Type |
| --- | --- | --- | --- | --- | --- | --- | --- | --- | --- | --- |
| 2 | 99 | EIVLTQSPGTLSLSPGER |  | cleaved G-E@N-term | 0.0032 | 1883.003 | 2 | 20 | 1.1.1.3663.21 | Winner |

85

| Contrib | Conf | Sequence | Modifications | Cleavages | ∆Mass | Prec MW | z | Sc | Spectrum | Type |
| --- | --- | --- | --- | --- | --- | --- | --- | --- | --- | --- |
| 1.38 | 99 | LGNQEPGGQTALK |  |  | 0.0005 | 1311.679 | 2 | 13 | 1.1.1.2720.23 | Winner |

TSP (PEG4000)-solution-rep 2

67

| Contrib | Conf | Sequence | Modifications | Cleavages | ∆Mass | Prec MW | z | Sc | Spectrum | Type |
| --- | --- | --- | --- | --- | --- | --- | --- | --- | --- | --- |
| 2 | 99 | LFDSDPITVTVPVEVSR |  |  | 0.003 | 1872.986 | 2 | 22 | 1.1.1.3927.31 | Winner |

68

| Contrib | Conf | Sequence | Modifications | Cleavages | ∆Mass | Prec MW | z | Sc | Spectrum | Type |
| --- | --- | --- | --- | --- | --- | --- | --- | --- | --- | --- |
| 2 | 99 | EPFLSCCQFAESLR | Carbamidomethyl(C)@6; Carbamidomethyl(C)@7 |  | -0.0043 | 1742.771 | 2 | 16 | 1.1.1.3832.21 | Winner |

69

| Contrib | Conf | Sequence | Modifications | Cleavages | ∆Mass | Prec MW | z | Sc | Spectrum | Type |
| --- | --- | --- | --- | --- | --- | --- | --- | --- | --- | --- |
| 2 | 99 | FLVGPDGIPIMR |  |  | 0.0008 | 1313.718 | 2 | 11 | 1.1.1.3930.9 | Winner |

70

| Contrib | Conf | Sequence | Modifications | Cleavages | ∆Mass | Prec MW | z | Sc | Spectrum | Type |
| --- | --- | --- | --- | --- | --- | --- | --- | --- | --- | --- |
| 2 | 99 | TPEVTCVVVDVSHEDPEVQFK | Carbamidomethyl(C)@6 |  | 0.0034 | 2413.151 | 3 | 17 | 1.1.1.3816.39 | Winner |

71

| Contrib | Conf | Sequence | Modifications | Cleavages | ∆Mass | Prec MW | z | Sc | Spectrum | Type |
| --- | --- | --- | --- | --- | --- | --- | --- | --- | --- | --- |
| 2 | 99 | DASGATFTWTPSSGK |  |  | 0.0022 | 1511.692 | 2 | 21 | 1.1.1.3412.20 | Winner |

72

| Contrib | Conf | Sequence | Modifications | Cleavages | ∆Mass | Prec MW | z | Sc | Spectrum | Type |
| --- | --- | --- | --- | --- | --- | --- | --- | --- | --- | --- |
| 2 | 99 | AAPSVTLFPPSSEELQANK |  |  | -0.003 | 1985.007 | 2 | 16 | 1.1.1.3653.17 | Other |

73

| Contrib | Conf | Sequence | Modifications | Cleavages | ∆Mass | Prec MW | z | Sc | Spectrum | Type |
| --- | --- | --- | --- | --- | --- | --- | --- | --- | --- | --- |
| 2 | 99 | QVQLVQSGAEVK | Gln->pyro-Glu@N-term | cleaved S-Q@N-term | -0.0002 | 1267.677 | 2 | 20 | 1.1.1.3402.31 | Winner |

74

| Contrib | Conf | Sequence | Modifications | Cleavages | ∆Mass | Prec MW | z | Sc | Spectrum | Type |
| --- | --- | --- | --- | --- | --- | --- | --- | --- | --- | --- |
| 2 | 99 | VGAHAGEYGAEALER |  |  | 0.0034 | 1528.731 | 3 | 19 | 1.1.1.2920.6 | Winner |

75

| Contrib | Conf | Sequence | Modifications | Cleavages | ∆Mass | Prec MW | z | Sc | Spectrum | Type |
| --- | --- | --- | --- | --- | --- | --- | --- | --- | --- | --- |
| 2 | 99 | EVQLLESGGGLVQPGGSLR |  |  | -0.0066 | 1895.005 | 2 | 19 | 1.1.1.3707.18 | Winner |

76

| Contrib | Conf | Sequence | Modifications | Cleavages | ∆Mass | Prec MW | z | Sc | Spectrum | Type |
| --- | --- | --- | --- | --- | --- | --- | --- | --- | --- | --- |
| 2 | 99 | DIQMTQSPSTLSASVGDR |  |  | 0.0086 | 1891.903 | 2 | 12 | 1.1.1.3381.45 | Winner |

77

| Contrib | Conf | Sequence | Modifications | Cleavages | ∆Mass | Prec MW | z | Sc | Spectrum | Type |
| --- | --- | --- | --- | --- | --- | --- | --- | --- | --- | --- |
| 2 | 99 | DIQMTQSPSSLSASVGDR |  |  | 0.0044 | 1877.883 | 2 | 24 | 1.1.1.3372.15 | Winner |

78

| Contrib | Conf | Sequence | Modifications | Cleavages | ∆Mass | Prec MW | z | Sc | Spectrum | Type |
| --- | --- | --- | --- | --- | --- | --- | --- | --- | --- | --- |
| 2 | 99 | ILLDEQAQWK |  |  | 0.0019 | 1242.663 | 2 | 14 | 1.1.1.3473.9 | Winner |

79

| Contrib | Conf | Sequence | Modifications | Cleavages | ∆Mass | Prec MW | z | Sc | Spectrum | Type |
| --- | --- | --- | --- | --- | --- | --- | --- | --- | --- | --- |
| 2 | 99 | QSVLTQPPSVSGAPGQR | Gln->pyro-Glu@N-term |  | 0.0021 | 1690.866 | 2 | 15 | 1.1.1.3401.37 | Winner |

81

| Contrib | Conf | Sequence | Modifications | Cleavages | ∆Mass | Prec MW | z | Sc | Spectrum | Type |
| --- | --- | --- | --- | --- | --- | --- | --- | --- | --- | --- |
| 1.8 | 99 | ADGLAVIGVLMK |  |  | -0.0006 | 1185.679 | 2 | 16 | 1.1.1.4139.7 | Winner |

TSP (PEG6000)-solution-rep 1

82

| Contrib | Conf | Sequence | Modifications | Cleavages | ∆Mass | Prec MW | z | Sc | Spectrum | Type |
| --- | --- | --- | --- | --- | --- | --- | --- | --- | --- | --- |
| 2 | 99 | TPEVTCVVVDVSHEDPEVQFK | Carbamidomethyl(C)@6 |  | 0.0037 | 2413.151 | 3 | 20 | 1.1.1.3776.30 | Winner |

83

| Contrib | Conf | Sequence | Modifications | Cleavages | ∆Mass | Prec MW | z | Sc | Spectrum | Type |
| --- | --- | --- | --- | --- | --- | --- | --- | --- | --- | --- |
| 2 | 99 | AAPSVTLFPPSSEELQANK |  |  | -0.0054 | 1985.005 | 2 | 15 | 1.1.1.3674.19 | Winner |

84

| Contrib | Conf | Sequence | Modifications | Cleavages | ∆Mass | Prec MW | z | Sc | Spectrum | Type |
| --- | --- | --- | --- | --- | --- | --- | --- | --- | --- | --- |
| 2 | 99 | VLLESIGSHEELTQDSR |  |  | 0.0073 | 1911.961 | 3 | 13 | 1.1.1.3352.11 | Winner |

85

| Contrib | Conf | Sequence | Modifications | Cleavages | ∆Mass | Prec MW | z | Sc | Spectrum | Type |
| --- | --- | --- | --- | --- | --- | --- | --- | --- | --- | --- |
| 2 | 99 | LTLQQKEHEQKMQLLLHHFK |  | missed K-E@6; missed K-M@11 | -0.0094 | 2528.359 | 4 | 10 | 1.1.1.3993.3 | Winner |

86

| Contrib | Conf | Sequence | Modifications | Cleavages | ∆Mass | Prec MW | z | Sc | Spectrum | Type |
| --- | --- | --- | --- | --- | --- | --- | --- | --- | --- | --- |
| 2 | 99 | EPLGATIR | Glu->pyro-Glu@N-term |  | -0.0012 | 837.4697 | 2 | 7 | 1.1.1.3955.2 | Winner |

87

| Contrib | Conf | Sequence | Modifications | Cleavages | ∆Mass | Prec MW | z | Sc | Spectrum | Type |
| --- | --- | --- | --- | --- | --- | --- | --- | --- | --- | --- |
| 2 | 99 | QEALELMNQNLDIYEQQVMTAAQK |  |  | -0.0062 | 2807.341 | 3 | 11 | 1.1.1.4266.35 | Winner |

88

| Contrib | Conf | Sequence | Modifications | Cleavages | ∆Mass | Prec MW | z | Sc | Spectrum | Type |
| --- | --- | --- | --- | --- | --- | --- | --- | --- | --- | --- |
| 2 | 99 | GGPQPTPSPAGPGTQLGPITGEMDEADSAFLK | Oxidation(M)@23 |  | 0.0072 | 3138.489 | 4 | 11 | 1.1.1.3974.10 | Winner |

89

| Contrib | Conf | Sequence | Modifications | Cleavages | ∆Mass | Prec MW | z | Sc | Spectrum | Type |
| --- | --- | --- | --- | --- | --- | --- | --- | --- | --- | --- |
| 2 | 99 | ALAQCAPPPAVCAELVR | Carbamidomethyl(C)@5; Carbamidomethyl(C)@12 |  | 0.0011 | 1821.924 | 3 | 19 | 1.1.1.3445.10 | Winner |

90

| Contrib | Conf | Sequence | Modifications | Cleavages | ∆Mass | Prec MW | z | Sc | Spectrum | Type |
| --- | --- | --- | --- | --- | --- | --- | --- | --- | --- | --- |
| 2 | 99 | VPLALFALNR |  |  | 0.0075 | 1112.678 | 2 | 9 | 1.1.1.3915.5 | Winner |

91

| Contrib | Conf | Sequence | Modifications | Cleavages | ∆Mass | Prec MW | z | Sc | Spectrum | Type |
| --- | --- | --- | --- | --- | --- | --- | --- | --- | --- | --- |
| 2 | 99 | QVQLVQSGAEVK | Gln->pyro-Glu@N-term |  | 0.0024 | 1267.68 | 2 | 16 | 1.1.1.3380.8 | Winner |

92

| Contrib | Conf | Sequence | Modifications | Cleavages | ∆Mass | Prec MW | z | Sc | Spectrum | Type |
| --- | --- | --- | --- | --- | --- | --- | --- | --- | --- | --- |
| 2 | 99 | EVQLLESGGGLVQPGGSLR |  | cleaved C-E@N-term | -0.011 | 1895 | 2 | 19 | 1.1.1.3677.20 | Winner |

93

| Contrib | Conf | Sequence | Modifications | Cleavages | ∆Mass | Prec MW | z | Sc | Spectrum | Type |
| --- | --- | --- | --- | --- | --- | --- | --- | --- | --- | --- |
| 2 | 99 | TIAVLLDDILQR |  |  | 0.0012 | 1368.799 | 2 | 13 | 1.1.1.4360.6 | Winner |

94

| Contrib | Conf | Sequence | Modifications | Cleavages | ∆Mass | Prec MW | z | Sc | Spectrum | Type |
| --- | --- | --- | --- | --- | --- | --- | --- | --- | --- | --- |
| 2 | 99 | IFYNQQNHYDGSTGK |  |  | 0.0044 | 1770.801 | 3 | 13 | 1.1.1.2819.19 | Winner |

95

| Contrib | Conf | Sequence | Modifications | Cleavages | ∆Mass | Prec MW | z | Sc | Spectrum | Type |
| --- | --- | --- | --- | --- | --- | --- | --- | --- | --- | --- |
| 2 | 99 | VADFLSWCR | Carbamidomethyl(C)@8 |  | 0.0081 | 1152.547 | 2 | 9 | 1.1.1.3770.6 | Winner |

96

| Contrib | Conf | Sequence | Modifications | Cleavages | ∆Mass | Prec MW | z | Sc | Spectrum | Type |
| --- | --- | --- | --- | --- | --- | --- | --- | --- | --- | --- |
| 2 | 99 | FLVGPDGIPIMR |  |  | 0.0052 | 1313.722 | 2 | 13 | 1.1.1.3892.10 | Winner |

97

| Contrib | Conf | Sequence | Modifications | Cleavages | ∆Mass | Prec MW | z | Sc | Spectrum | Type |
| --- | --- | --- | --- | --- | --- | --- | --- | --- | --- | --- |
| 2 | 99 | IPLNDLFR |  |  | 0.0015 | 986.5565 | 2 | 7 | 1.1.1.3737.3 | Winner |

98

| Contrib | Conf | Sequence | Modifications | Cleavages | ∆Mass | Prec MW | z | Sc | Spectrum | Type |
| --- | --- | --- | --- | --- | --- | --- | --- | --- | --- | --- |
| 2 | 99 | DIQMTQSPSSLSASVGDR |  |  | 0.0053 | 1877.884 | 2 | 24 | 1.1.1.3349.17 | Winner |

99

| Contrib | Conf | Sequence | Modifications | Cleavages | ∆Mass | Prec MW | z | Sc | Spectrum | Type |
| --- | --- | --- | --- | --- | --- | --- | --- | --- | --- | --- |
| 2 | 99 | LTVLSQPK |  |  | -0.0004 | 884.5327 | 2 | 10 | 1.1.1.2839.6 | Winner |

100

| Contrib | Conf | Sequence | Modifications | Cleavages | ∆Mass | Prec MW | z | Sc | Spectrum | Type |
| --- | --- | --- | --- | --- | --- | --- | --- | --- | --- | --- |
| 2 | 99 | QSVLTQPPSVSGAPGQR | Gln->pyro-Glu@N-term |  | 0.0032 | 1690.867 | 2 | 14 | 1.1.1.3374.17 | Winner |

TSP (PEG6000)-solution-rep 2

78

| Contrib | Conf | Sequence | Modifications | Cleavages | ∆Mass | Prec MW | z | Sc | Spectrum | Type |
| --- | --- | --- | --- | --- | --- | --- | --- | --- | --- | --- |
| 2 | 99 | LSNNALSGLPQGVFGK |  |  | 0.0008 | 1600.858 | 2 | 13 | 1.1.1.4051.21 | Winner |

79

| Contrib | Conf | Sequence | Modifications | Cleavages | ∆Mass | Prec MW | z | Sc | Spectrum | Type |
| --- | --- | --- | --- | --- | --- | --- | --- | --- | --- | --- |
| 2 | 99 | TPEVTCVVVDVSHEDPEVQFK | Carbamidomethyl(C)@6 |  | 0.0001 | 2413.147 | 3 | 19 | 1.1.1.4071.33 | Winner |

80

| Contrib | Conf | Sequence | Modifications | Cleavages | ∆Mass | Prec MW | z | Sc | Spectrum | Type |
| --- | --- | --- | --- | --- | --- | --- | --- | --- | --- | --- |
| 2 | 99 | AAPSVTLFPPSSEELQANK |  |  | -0.0026 | 1985.008 | 2 | 18 | 1.1.1.3993.8 | Winner |

81

| Contrib | Conf | Sequence | Modifications | Cleavages | ∆Mass | Prec MW | z | Sc | Spectrum | Type |
| --- | --- | --- | --- | --- | --- | --- | --- | --- | --- | --- |
| 2 | 99 | VGALSQLR |  |  | -0.0015 | 842.4958 | 2 | 12 | 1.1.1.3150.5 | Winner |

82

| Contrib | Conf | Sequence | Modifications | Cleavages | ∆Mass | Prec MW | z | Sc | Spectrum | Type |
| --- | --- | --- | --- | --- | --- | --- | --- | --- | --- | --- |
| 2 | 99 | EYGVVLAPDGSTVAVEPLLAGLEAGLQGR |  |  | 0.0015 | 2880.525 | 3 | 15 | 1.1.1.4740.10 | Winner |

83

| Contrib | Conf | Sequence | Modifications | Cleavages | ∆Mass | Prec MW | z | Sc | Spectrum | Type |
| --- | --- | --- | --- | --- | --- | --- | --- | --- | --- | --- |
| 2 | 99 | LTVLSQPK |  | cleaved Q-L@N-term | 0.0001 | 884.5333 | 2 | 12 | 1.1.1.3128.3 | Winner |

84

| Contrib | Conf | Sequence | Modifications | Cleavages | ∆Mass | Prec MW | z | Sc | Spectrum | Type |
| --- | --- | --- | --- | --- | --- | --- | --- | --- | --- | --- |
| 2 | 99 | TIAVLLDDILQR |  |  | 0.004 | 1368.802 | 2 | 11 | 1.1.1.4641.6 | Winner |

85

| Contrib | Conf | Sequence | Modifications | Cleavages | ∆Mass | Prec MW | z | Sc | Spectrum | Type |
| --- | --- | --- | --- | --- | --- | --- | --- | --- | --- | --- |
| 1.96 | 99 | LSSGLVTAALYGR |  |  | 0.0021 | 1306.726 | 2 | 14 | 1.1.1.3962.18 | Winner |

86

| Contrib | Conf | Sequence | Modifications | Cleavages | ∆Mass | Prec MW | z | Sc | Spectrum | Type |
| --- | --- | --- | --- | --- | --- | --- | --- | --- | --- | --- |
| 1.92 | 99 | EVQLLESGGGLVQPGGSLR |  |  | -0.0042 | 1895.007 | 2 | 22 | 1.1.1.3966.13 | Winner |

87

| Contrib | Conf | Sequence | Modifications | Cleavages | ∆Mass | Prec MW | z | Sc | Spectrum | Type |
| --- | --- | --- | --- | --- | --- | --- | --- | --- | --- | --- |
| 1.77 | 99 | FLVGPDGIPIMR |  |  | 0.0008 | 1313.718 | 2 | 11 | 1.1.1.4179.15 | Winner |

88

| Contrib | Conf | Sequence | Modifications | Cleavages | ∆Mass | Prec MW | z | Sc | Spectrum | Type |
| --- | --- | --- | --- | --- | --- | --- | --- | --- | --- | --- |
| 1.64 | 99 | QFTSSTSYNR |  |  | 0.0008 | 1189.537 | 2 | 11 | 1.1.1.2968.14 | Winner |

89

| Contrib | Conf | Sequence | Modifications | Cleavages | ∆Mass | Prec MW | z | Sc | Spectrum | Type |
| --- | --- | --- | --- | --- | --- | --- | --- | --- | --- | --- |
| 1.47 | 99 | MPGTVATLR |  |  | -0.001 | 944.5105 | 2 | 10 | 1.1.1.3313.6 | Winner |

90

| Contrib | Conf | Sequence | Modifications | Cleavages | ∆Mass | Prec MW | z | Sc | Spectrum | Type |
| --- | --- | --- | --- | --- | --- | --- | --- | --- | --- | --- |
| 1.38 | 99 | VLEDNSALDK |  |  | 0.0049 | 1102.555 | 2 | 12 | 1.1.1.3038.9 | Winner |

92

| Contrib | Conf | Sequence | Modifications | Cleavages | ∆Mass | Prec MW | z | Sc | Spectrum | Type |
| --- | --- | --- | --- | --- | --- | --- | --- | --- | --- | --- |
| 1.38 | 99 | VLLESIGSHEELTQDSR |  |  | -0.001 | 1911.953 | 3 | 17 | 1.1.1.3633.9 | Winner |

93

| Contrib | Conf | Sequence | Modifications | Cleavages | ∆Mass | Prec MW | z | Sc | Spectrum | Type |
| --- | --- | --- | --- | --- | --- | --- | --- | --- | --- | --- |
| 1.37 | 99 | VQLTGEPVPMAR |  |  | 0.0026 | 1296.689 | 2 | 13 | 1.1.1.3481.20 | Winner |

94

| Contrib | Conf | Sequence | Modifications | Cleavages | ∆Mass | Prec MW | z | Sc | Spectrum | Type |
| --- | --- | --- | --- | --- | --- | --- | --- | --- | --- | --- |
| 1.37 | 99 | FLPEGCQPLVSSAVDR | Carbamidomethyl(C)@6 |  | 0.0016 | 1773.874 | 2 | 14 | 1.1.1.3760.35 | Winner |

95

| Contrib | Conf | Sequence | Modifications | Cleavages | ∆Mass | Prec MW | z | Sc | Spectrum | Type |
| --- | --- | --- | --- | --- | --- | --- | --- | --- | --- | --- |
| 1.37 | 99 | DIQMTQSPSSLSASVGDR |  | cleaved C-D@N-term | 0.0028 | 1877.882 | 2 | 19 | 1.1.1.3632.17 | Winner |

96

| Contrib | Conf | Sequence | Modifications | Cleavages | ∆Mass | Prec MW | z | Sc | Spectrum | Type |
| --- | --- | --- | --- | --- | --- | --- | --- | --- | --- | --- |
| 1.36 | 99 | ALAQCAPPPAVCAELVR | Carbamidomethyl(C)@5; Carbamidomethyl(C)@12 |  | 0.0021 | 1821.925 | 3 | 15 | 1.1.1.3729.10 | Winner |

97

| Contrib | Conf | Sequence | Modifications | Cleavages | ∆Mass | Prec MW | z | Sc | Spectrum | Type |
| --- | --- | --- | --- | --- | --- | --- | --- | --- | --- | --- |
| 1.36 | 99 | EVQLVESGGGLVQPGGSLR |  |  | -0.0079 | 1880.988 | 3 | 19 | 1.1.1.3798.7 | Winner |

FC-TSP (PEG4000)-solution-rep 1

100

| Contrib | Conf | Sequence | Modifications | Cleavages | ∆Mass | Prec MW | z | Sc | Spectrum | Type |
| --- | --- | --- | --- | --- | --- | --- | --- | --- | --- | --- |
| 2 | 99 | EALQGVGDMGR |  |  | -0.0025 | 1131.532 | 2 | 16 | 1.1.1.2871.12 | Winner |

101

| Contrib | Conf | Sequence | Modifications | Cleavages | ∆Mass | Prec MW | z | Sc | Spectrum | Type |
| --- | --- | --- | --- | --- | --- | --- | --- | --- | --- | --- |
| 2 | 99 | AEELSIQVSCR | Carbamidomethyl(C)@10 |  | -0.0003 | 1290.623 | 2 | 13 | 1.1.1.3032.31 | Winner |

102

| Contrib | Conf | Sequence | Modifications | Cleavages | ∆Mass | Prec MW | z | Sc | Spectrum | Type |
| --- | --- | --- | --- | --- | --- | --- | --- | --- | --- | --- |
| 2 | 99 | SYDLDPGAGSLEI |  |  | -0.0002 | 1335.619 | 2 | 17 | 1.1.1.3814.7 | Winner |

103

| Contrib | Conf | Sequence | Modifications | Cleavages | ∆Mass | Prec MW | z | Sc | Spectrum | Type |
| --- | --- | --- | --- | --- | --- | --- | --- | --- | --- | --- |
| 2 | 99 | NSCPPTSELLGTSDR | Carbamidomethyl(C)@3 |  | 0.0021 | 1632.744 | 2 | 16 | 1.1.1.3039.36 | Winner |

104

| Contrib | Conf | Sequence | Modifications | Cleavages | ∆Mass | Prec MW | z | Sc | Spectrum | Type |
| --- | --- | --- | --- | --- | --- | --- | --- | --- | --- | --- |
| 2 | 99 | TPEVTCVVVDVSHEDPEVQFK | Carbamidomethyl(C)@6 |  | 0.0009 | 2413.148 | 3 | 16 | 1.1.1.3702.27 | Winner |

105

| Contrib | Conf | Sequence | Modifications | Cleavages | ∆Mass | Prec MW | z | Sc | Spectrum | Type |
| --- | --- | --- | --- | --- | --- | --- | --- | --- | --- | --- |
| 2 | 99 | DASGATFTWTPSSGK |  |  | 0.0017 | 1511.691 | 2 | 20 | 1.1.1.3317.13 | Winner |

106

| Contrib | Conf | Sequence | Modifications | Cleavages | ∆Mass | Prec MW | z | Sc | Spectrum | Type |
| --- | --- | --- | --- | --- | --- | --- | --- | --- | --- | --- |
| 2 | 99 | AEMADQAAAWLTR |  |  | -0.001 | 1432.676 | 2 | 16 | 1.1.1.3596.13 | Winner |

108

| Contrib | Conf | Sequence | Modifications | Cleavages | ∆Mass | Prec MW | z | Sc | Spectrum | Type |
| --- | --- | --- | --- | --- | --- | --- | --- | --- | --- | --- |
| 2 | 99 | ALAQCAPPPAVCAELVR | Carbamidomethyl(C)@5; Carbamidomethyl(C)@12 |  | -0.0014 | 1821.922 | 3 | 16 | 1.1.1.3372.4 | Winner |

109

| Contrib | Conf | Sequence | Modifications | Cleavages | ∆Mass | Prec MW | z | Sc | Spectrum | Type |
| --- | --- | --- | --- | --- | --- | --- | --- | --- | --- | --- |
| 2 | 99 | FFYEFSDSSLSK |  |  | -0.0003 | 1455.656 | 2 | 12 | 1.1.1.2855.35 | Winner |

110

| Contrib | Conf | Sequence | Modifications | Cleavages | ∆Mass | Prec MW | z | Sc | Spectrum | Type |
| --- | --- | --- | --- | --- | --- | --- | --- | --- | --- | --- |
| 2 | 99 | DIQMTQSPSSLSASVGDR |  | cleaved C-D@N-term | 0.0002 | 1877.879 | 2 | 17 | 1.1.1.3269.25 | Winner |

111

| Contrib | Conf | Sequence | Modifications | Cleavages | ∆Mass | Prec MW | z | Sc | Spectrum | Type |
| --- | --- | --- | --- | --- | --- | --- | --- | --- | --- | --- |
| 2 | 99 | QVQLVQSGAEVK | Deamidated(Q)@1 | cleaved S-Q@N-term | 0.0001 | 1285.688 | 2 | 14 | 1.1.1.2865.16 | Winner |

112

| Contrib | Conf | Sequence | Modifications | Cleavages | ∆Mass | Prec MW | z | Sc | Spectrum | Type |
| --- | --- | --- | --- | --- | --- | --- | --- | --- | --- | --- |
| 2 | 99 | LTAFPSESVK |  |  | -0.0026 | 1077.568 | 2 | 10 | 1.1.1.2986.20 | Winner |

113

| Contrib | Conf | Sequence | Modifications | Cleavages | ∆Mass | Prec MW | z | Sc | Spectrum | Type |
| --- | --- | --- | --- | --- | --- | --- | --- | --- | --- | --- |
| 2 | 99 | SCDVESNPGIFLPPGTQAEFNLR | Carbamidomethyl(C)@2 |  | -0.0052 | 2547.201 | 3 | 17 | 1.1.1.3976.16 | Winner |

114

| Contrib | Conf | Sequence | Modifications | Cleavages | ∆Mass | Prec MW | z | Sc | Spectrum | Type |
| --- | --- | --- | --- | --- | --- | --- | --- | --- | --- | --- |
| 2 | 99 | EVQLLESGGGLVQPGGSLR |  |  | 0.0005 | 1895.012 | 2 | 21 | 1.1.1.3593.37 | Winner |

115

| Contrib | Conf | Sequence | Modifications | Cleavages | ∆Mass | Prec MW | z | Sc | Spectrum | Type |
| --- | --- | --- | --- | --- | --- | --- | --- | --- | --- | --- |
| 2 | 99 | SYELTQPPSVSVSPGQTAR |  |  | -0.0037 | 2002.992 | 2 | 14 | 1.1.1.3273.28 | Winner |

116

| Contrib | Conf | Sequence | Modifications | Cleavages | ∆Mass | Prec MW | z | Sc | Spectrum | Type |
| --- | --- | --- | --- | --- | --- | --- | --- | --- | --- | --- |
| 2 | 99 | FSGSGSGTDFTLK |  |  | 0.0056 | 1302.615 | 2 | 13 | 1.1.1.3077.20 | Winner |

117

| Contrib | Conf | Sequence | Modifications | Cleavages | ∆Mass | Prec MW | z | Sc | Spectrum | Type |
| --- | --- | --- | --- | --- | --- | --- | --- | --- | --- | --- |
| 2 | 99 | DIQMTQSPSTLSASVGDR |  | cleaved C-D@N-term | -0.0037 | 1891.891 | 2 | 15 | 1.1.1.3275.18 | Winner |

118

| Contrib | Conf | Sequence | Modifications | Cleavages | ∆Mass | Prec MW | z | Sc | Spectrum | Type |
| --- | --- | --- | --- | --- | --- | --- | --- | --- | --- | --- |
| 2 | 99 | TGDIVEFVCK | Carbamidomethyl(C)@9 |  | -0.0002 | 1166.564 | 2 | 12 | 1.1.1.3361.12 | Winner |

119

| Contrib | Conf | Sequence | Modifications | Cleavages | ∆Mass | Prec MW | z | Sc | Spectrum | Type |
| --- | --- | --- | --- | --- | --- | --- | --- | --- | --- | --- |
| 2 | 99 | GIVEECCFR | Carbamidomethyl(C)@6; Carbamidomethyl(C)@7 |  | 0.0031 | 1168.504 | 2 | 10 | 1.1.1.2986.25 | Winner |

120

| Contrib | Conf | Sequence | Modifications | Cleavages | ∆Mass | Prec MW | z | Sc | Spectrum | Type |
| --- | --- | --- | --- | --- | --- | --- | --- | --- | --- | --- |
| 2 | 99 | ALDFAVGEYNK |  |  | -0.001 | 1225.597 | 2 | 13 | 1.1.1.3378.23 | Winner |

121

| Contrib | Conf | Sequence | Modifications | Cleavages | ∆Mass | Prec MW | z | Sc | Spectrum | Type |
| --- | --- | --- | --- | --- | --- | --- | --- | --- | --- | --- |
| 1.66 | 99 | FAALDNEEEDK |  |  | -0.0092 | 1279.548 | 2 | 11 | 1.1.1.2769.25 | Winner |

122

| Contrib | Conf | Sequence | Modifications | Cleavages | ∆Mass | Prec MW | z | Sc | Spectrum | Type |
| --- | --- | --- | --- | --- | --- | --- | --- | --- | --- | --- |
| 1.43 | 99 | LVEVLK |  | cleaved V-L@N-term | -0.0004 | 699.4527 | 2 | 8 | 1.1.1.2941.2 | Winner |

FC-TSP (PEG4000)-solution-rep 2

102

| Contrib | Conf | Sequence | Modifications | Cleavages | ∆Mass | Prec MW | z | Sc | Spectrum | Type |
| --- | --- | --- | --- | --- | --- | --- | --- | --- | --- | --- |
| 2 | 99 | EALQGVGDMGR |  |  | -0.0018 | 1131.533 | 2 | 12 | 1.1.1.2837.9 | Winner |

103

| Contrib | Conf | Sequence | Modifications | Cleavages | ∆Mass | Prec MW | z | Sc | Spectrum | Type |
| --- | --- | --- | --- | --- | --- | --- | --- | --- | --- | --- |
| 2 | 99 | EIMENYNIALR |  |  | 0.0021 | 1364.678 | 2 | 15 | 1.1.1.3410.14 | Winner |

104

| Contrib | Conf | Sequence | Modifications | Cleavages | ∆Mass | Prec MW | z | Sc | Spectrum | Type |
| --- | --- | --- | --- | --- | --- | --- | --- | --- | --- | --- |
| 2 | 99 | DASGATFTWTPSSGK |  |  | -0.0006 | 1511.689 | 2 | 23 | 1.1.1.3282.13 | Winner |

106

| Contrib | Conf | Sequence | Modifications | Cleavages | ∆Mass | Prec MW | z | Sc | Spectrum | Type |
| --- | --- | --- | --- | --- | --- | --- | --- | --- | --- | --- |
| 2 | 99 | SYELPDGQVITIGNER |  |  | 0.0032 | 1789.888 | 2 | 14 | 1.1.1.3694.23 | Winner |

107

| Contrib | Conf | Sequence | Modifications | Cleavages | ∆Mass | Prec MW | z | Sc | Spectrum | Type |
| --- | --- | --- | --- | --- | --- | --- | --- | --- | --- | --- |
| 2 | 99 | GCPAALPLSNLYETLGVVGSTTTQLYTDR | Carbamidomethyl(C)@2 |  | -0.0003 | 3096.542 | 3 | 19 | 1.1.1.4516.14 | Winner |

108

| Contrib | Conf | Sequence | Modifications | Cleavages | ∆Mass | Prec MW | z | Sc | Spectrum | Type |
| --- | --- | --- | --- | --- | --- | --- | --- | --- | --- | --- |
| 2 | 99 | EVGPPLPQEAVPLQK |  |  | 0.0031 | 1600.886 | 2 | 15 | 1.1.1.3353.23 | Winner |

109

| Contrib | Conf | Sequence | Modifications | Cleavages | ∆Mass | Prec MW | z | Sc | Spectrum | Type |
| --- | --- | --- | --- | --- | --- | --- | --- | --- | --- | --- |
| 2 | 99 | FSIEGSYQLEK |  |  | -0.0026 | 1299.632 | 2 | 12 | 1.1.1.3376.12 | Winner |

110

| Contrib | Conf | Sequence | Modifications | Cleavages | ∆Mass | Prec MW | z | Sc | Spectrum | Type |
| --- | --- | --- | --- | --- | --- | --- | --- | --- | --- | --- |
| 2 | 99 | VDAETGDVFAIER |  |  | -0.0041 | 1420.679 | 2 | 15 | 1.1.1.3390.17 | Winner |

111

| Contrib | Conf | Sequence | Modifications | Cleavages | ∆Mass | Prec MW | z | Sc | Spectrum | Type |
| --- | --- | --- | --- | --- | --- | --- | --- | --- | --- | --- |
| 2 | 99 | FFYEFSDSSLSK |  |  | -0.0013 | 1455.654 | 2 | 12 | 1.1.1.2831.15 | Winner |

112

| Contrib | Conf | Sequence | Modifications | Cleavages | ∆Mass | Prec MW | z | Sc | Spectrum | Type |
| --- | --- | --- | --- | --- | --- | --- | --- | --- | --- | --- |
| 2 | 99 | MESLGITSR |  |  | -0.0022 | 992.4939 | 2 | 9 | 1.1.1.2842.4 | Winner |

113

| Contrib | Conf | Sequence | Modifications | Cleavages | ∆Mass | Prec MW | z | Sc | Spectrum | Type |
| --- | --- | --- | --- | --- | --- | --- | --- | --- | --- | --- |
| 2 | 99 | IADQCPSSLAIQENANALAR | Carbamidomethyl(C)@5 |  | 0.0041 | 2141.058 | 3 | 15 | 1.1.1.3353.21 | Winner |

114

| Contrib | Conf | Sequence | Modifications | Cleavages | ∆Mass | Prec MW | z | Sc | Spectrum | Type |
| --- | --- | --- | --- | --- | --- | --- | --- | --- | --- | --- |
| 2 | 99 | TGDIVEFVCK | Carbamidomethyl(C)@9 |  | 0.0018 | 1166.566 | 2 | 12 | 1.1.1.3330.7 | Winner |

115

| Contrib | Conf | Sequence | Modifications | Cleavages | ∆Mass | Prec MW | z | Sc | Spectrum | Type |
| --- | --- | --- | --- | --- | --- | --- | --- | --- | --- | --- |
| 2 | 99 | TITLEVEPSDTIENVK |  |  | -0.0024 | 1786.918 | 2 | 12 | 1.1.1.3458.36 | Winner |

116

| Contrib | Conf | Sequence | Modifications | Cleavages | ∆Mass | Prec MW | z | Sc | Spectrum | Type |
| --- | --- | --- | --- | --- | --- | --- | --- | --- | --- | --- |
| 2 | 99 | TGVITSPDFPNPYPK |  |  | 0.0004 | 1631.82 | 2 | 15 | 1.1.1.3416.38 | Winner |

117

| Contrib | Conf | Sequence | Modifications | Cleavages | ∆Mass | Prec MW | z | Sc | Spectrum | Type |
| --- | --- | --- | --- | --- | --- | --- | --- | --- | --- | --- |
| 2 | 99 | FLVGPDGIPIMR |  |  | 0.0021 | 1313.719 | 2 | 15 | 1.1.1.3821.6 | Winner |

118

| Contrib | Conf | Sequence | Modifications | Cleavages | ∆Mass | Prec MW | z | Sc | Spectrum | Type |
| --- | --- | --- | --- | --- | --- | --- | --- | --- | --- | --- |
| 2 | 99 | DIQMTQSPSSLSASVGDR |  | cleaved C-D@N-term | 0.0013 | 1877.88 | 2 | 23 | 1.1.1.3244.43 | Winner |

119

| Contrib | Conf | Sequence | Modifications | Cleavages | ∆Mass | Prec MW | z | Sc | Spectrum | Type |
| --- | --- | --- | --- | --- | --- | --- | --- | --- | --- | --- |
| 2 | 99 | SYELTQPPSVSVSPGQTAR |  |  | -0.0013 | 2002.995 | 3 | 16 | 1.1.1.3247.21 | Winner |

120

| Contrib | Conf | Sequence | Modifications | Cleavages | ∆Mass | Prec MW | z | Sc | Spectrum | Type |
| --- | --- | --- | --- | --- | --- | --- | --- | --- | --- | --- |
| 2 | 99 | FSGSGSGTDFTLK |  |  | 0.0042 | 1302.614 | 2 | 14 | 1.1.1.3019.10 | Winner |

121

| Contrib | Conf | Sequence | Modifications | Cleavages | ∆Mass | Prec MW | z | Sc | Spectrum | Type |
| --- | --- | --- | --- | --- | --- | --- | --- | --- | --- | --- |
| 2 | 99 | DIQMTQSPSTLSASVGDR |  | cleaved C-D@N-term | -0.0056 | 1891.889 | 2 | 22 | 1.1.1.3252.19 | Winner |

122

| Contrib | Conf | Sequence | Modifications | Cleavages | ∆Mass | Prec MW | z | Sc | Spectrum | Type |
| --- | --- | --- | --- | --- | --- | --- | --- | --- | --- | --- |
| 2 | 99 | NLILAGVK |  |  | 0.002 | 826.5297 | 2 | 9 | 1.1.1.3037.2 | Winner |

123

| Contrib | Conf | Sequence | Modifications | Cleavages | ∆Mass | Prec MW | z | Sc | Spectrum | Type |
| --- | --- | --- | --- | --- | --- | --- | --- | --- | --- | --- |
| 2 | 99 | EIVLTQSPGTLSLSPGER |  | cleaved G-E@N-term | -0.0008 | 1882.999 | 2 | 13 | 1.1.1.3464.46 | Winner |

124

| Contrib | Conf | Sequence | Modifications | Cleavages | ∆Mass | Prec MW | z | Sc | Spectrum | Type |
| --- | --- | --- | --- | --- | --- | --- | --- | --- | --- | --- |
| 1.96 | 99 | QSVLTQPPSVSGAPGQR | Gln->pyro-Glu@N-term |  | 0.0018 | 1690.866 | 2 | 10 | 1.1.1.3267.42 | Winner |

125

| Contrib | Conf | Sequence | Modifications | Cleavages | ∆Mass | Prec MW | z | Sc | Spectrum | Type |
| --- | --- | --- | --- | --- | --- | --- | --- | --- | --- | --- |
| 1.55 | 99 | NKAEEAMATYR | Deamidated(N)@1 | missed K-A@2 | -0.0146 | 1283.567 | 2 | 12 | 1.1.1.3351.12 | Winner |

FC-TSP (PEG6000)-solution-rep 1

102

| Contrib | Conf | Sequence | Modifications | Cleavages | ∆Mass | Prec MW | z | Sc | Spectrum | Type |
| --- | --- | --- | --- | --- | --- | --- | --- | --- | --- | --- |
| 2 | 99 | STSESTAALGCLVK | Carbamidomethyl(C)@11 |  | 0.0066 | 1422.709 | 2 | 19 | 1.1.1.3209.7 | Winner |

103

| Contrib | Conf | Sequence | Modifications | Cleavages | ∆Mass | Prec MW | z | Sc | Spectrum | Type |
| --- | --- | --- | --- | --- | --- | --- | --- | --- | --- | --- |
| 2 | 99 | LVGITSWGEGCAR | Carbamidomethyl(C)@11 |  | 0.0019 | 1404.684 | 2 | 11 | 1.1.1.3441.25 | Winner |

104

| Contrib | Conf | Sequence | Modifications | Cleavages | ∆Mass | Prec MW | z | Sc | Spectrum | Type |
| --- | --- | --- | --- | --- | --- | --- | --- | --- | --- | --- |
| 2 | 99 | DASGATFTWTPSSGK |  |  | -0.0013 | 1511.688 | 2 | 16 | 1.1.1.3311.23 | Winner |

107

| N | Unused | Total | % Cov | Accession # | Name | Species | Peptides(95%) | Biological Processes | Molecular Functions | PANTHER ID |
| --- | --- | --- | --- | --- | --- | --- | --- | --- | --- | --- |
| 107 | 2 | 2 | 4.9 | sp|A8TX70|CO6A5_HUMAN | Collagen alpha-5(VI) chain OS=Homo sapiens GN=COL6A5 PE=1 SV=1 | HUMAN | 1 |  |  |  |

108

| Contrib | Conf | Sequence | Modifications | Cleavages | ∆Mass | Prec MW | z | Sc | Spectrum | Type |
| --- | --- | --- | --- | --- | --- | --- | --- | --- | --- | --- |
| 2 | 99 | QVLLVGAPTYDDVSK |  |  | 0.0004 | 1603.846 | 2 | 11 | 1.1.1.3435.21 | Winner |

109

| Contrib | Conf | Sequence | Modifications | Cleavages | ∆Mass | Prec MW | z | Sc | Spectrum | Type |
| --- | --- | --- | --- | --- | --- | --- | --- | --- | --- | --- |
| 2 | 99 | DIQMTQSPSSLSASVGDR |  | cleaved C-D@N-term | 0.0072 | 1877.886 | 2 | 23 | 1.1.1.3260.12 | Winner |

110

| Contrib | Conf | Sequence | Modifications | Cleavages | ∆Mass | Prec MW | z | Sc | Spectrum | Type |
| --- | --- | --- | --- | --- | --- | --- | --- | --- | --- | --- |
| 2 | 99 | EVQLLESGGGLVQPGGSLR |  |  | 0.0069 | 1895.018 | 2 | 14 | 1.1.1.3601.34 | Winner |

111

| Contrib | Conf | Sequence | Modifications | Cleavages | ∆Mass | Prec MW | z | Sc | Spectrum | Type |
| --- | --- | --- | --- | --- | --- | --- | --- | --- | --- | --- |
| 2 | 99 | DTGTYGFLLPER |  |  | -0.0025 | 1367.669 | 2 | 14 | 1.1.1.3710.9 | Winner |

112

| Contrib | Conf | Sequence | Modifications | Cleavages | ∆Mass | Prec MW | z | Sc | Spectrum | Type |
| --- | --- | --- | --- | --- | --- | --- | --- | --- | --- | --- |
| 2 | 99 | AGPHCPTAQLIATLK | Carbamidomethyl(C)@5 |  | 0.0002 | 1576.84 | 3 | 9 | 1.1.1.3370.13 | Winner |

113

| Contrib | Conf | Sequence | Modifications | Cleavages | ∆Mass | Prec MW | z | Sc | Spectrum | Type |
| --- | --- | --- | --- | --- | --- | --- | --- | --- | --- | --- |
| 2 | 99 | QVQLVQSGAEVK | Gln->pyro-Glu@N-term |  | -0.0008 | 1267.677 | 2 | 19 | 1.1.1.3289.24 | Winner |

114

| Contrib | Conf | Sequence | Modifications | Cleavages | ∆Mass | Prec MW | z | Sc | Spectrum | Type |
| --- | --- | --- | --- | --- | --- | --- | --- | --- | --- | --- |
| 2 | 99 | ALDFAVGEYNK |  |  | 0.0027 | 1225.601 | 2 | 12 | 1.1.1.3378.22 | Winner |

115

| Contrib | Conf | Sequence | Modifications | Cleavages | ∆Mass | Prec MW | z | Sc | Spectrum | Type |
| --- | --- | --- | --- | --- | --- | --- | --- | --- | --- | --- |
| 2 | 99 | EVQLVESGGGLVQPGGSLR |  |  | 0.001 | 1880.997 | 2 | 19 | 1.1.1.3439.25 | Winner |

116

| Contrib | Conf | Sequence | Modifications | Cleavages | ∆Mass | Prec MW | z | Sc | Spectrum | Type |
| --- | --- | --- | --- | --- | --- | --- | --- | --- | --- | --- |
| 1.52 | 99 | NLILAGVK |  |  | 0.0014 | 826.5291 | 2 | 8 | 1.1.1.3135.2 | Winner |

FC-TSP (PEG6000)-solution-rep 2

105

| Contrib | Conf | Sequence | Modifications | Cleavages | ∆Mass | Prec MW | z | Sc | Spectrum | Type |
| --- | --- | --- | --- | --- | --- | --- | --- | --- | --- | --- |
| 2 | 99 | FLVGPDGIPIMR |  |  | 0.0015 | 1313.718 | 2 | 14 | 1.1.1.3762.5 | Winner |

106

| Contrib | Conf | Sequence | Modifications | Cleavages | ∆Mass | Prec MW | z | Sc | Spectrum | Type |
| --- | --- | --- | --- | --- | --- | --- | --- | --- | --- | --- |
| 2 | 99 | ALAQCAPPPAVCAELVR | Carbamidomethyl(C)@5; Carbamidomethyl(C)@12 |  | -0.0021 | 1821.921 | 2 | 15 | 1.1.1.3317.13 | Winner |

107

| Contrib | Conf | Sequence | Modifications | Cleavages | ∆Mass | Prec MW | z | Sc | Spectrum | Type |
| --- | --- | --- | --- | --- | --- | --- | --- | --- | --- | --- |
| 2 | 99 | DASGATFTWTPSSGK |  |  | 0.003 | 1511.692 | 2 | 20 | 1.1.1.3259.16 | Winner |

109

| Contrib | Conf | Sequence | Modifications | Cleavages | ∆Mass | Prec MW | z | Sc | Spectrum | Type |
| --- | --- | --- | --- | --- | --- | --- | --- | --- | --- | --- |
| 2 | 99 | FFYEFSDSSLSK |  |  | -0.0014 | 1455.654 | 2 | 12 | 1.1.1.2792.13 | Winner |

110

| Contrib | Conf | Sequence | Modifications | Cleavages | ∆Mass | Prec MW | z | Sc | Spectrum | Type |
| --- | --- | --- | --- | --- | --- | --- | --- | --- | --- | --- |
| 2 | 99 | EVQLLESGGGLVQPGGSLR |  |  | 0.0029 | 1895.014 | 2 | 21 | 1.1.1.3545.36 | Winner |

111

| Contrib | Conf | Sequence | Modifications | Cleavages | ∆Mass | Prec MW | z | Sc | Spectrum | Type |
| --- | --- | --- | --- | --- | --- | --- | --- | --- | --- | --- |
| 2 | 99 | MPGTVATLR |  |  | -0.0016 | 944.5096 | 2 | 10 | 1.1.1.2842.6 | Winner |

112

| Contrib | Conf | Sequence | Modifications | Cleavages | ∆Mass | Prec MW | z | Sc | Spectrum | Type |
| --- | --- | --- | --- | --- | --- | --- | --- | --- | --- | --- |
| 2 | 99 | AEIEYLEK |  |  | -0.0006 | 993.5013 | 2 | 9 | 1.1.1.2920.8 | Winner |

113

| Contrib | Conf | Sequence | Modifications | Cleavages | ∆Mass | Prec MW | z | Sc | Spectrum | Type |
| --- | --- | --- | --- | --- | --- | --- | --- | --- | --- | --- |
| 2 | 99 | VIGSGCNLDSAR | Carbamidomethyl(C)@6 |  | -0.0106 | 1247.582 | 2 | 13 | 1.1.1.2644.13 | Winner |

114

| Contrib | Conf | Sequence | Modifications | Cleavages | ∆Mass | Prec MW | z | Sc | Spectrum | Type |
| --- | --- | --- | --- | --- | --- | --- | --- | --- | --- | --- |
| 2 | 99 | DIQMTQSPSSLSASVGDR |  |  | -0.0016 | 1877.877 | 2 | 20 | 1.1.1.3211.35 | Winner |

115

| Contrib | Conf | Sequence | Modifications | Cleavages | ∆Mass | Prec MW | z | Sc | Spectrum | Type |
| --- | --- | --- | --- | --- | --- | --- | --- | --- | --- | --- |
| 2 | 99 | QVQLVQSGAEVK | Gln->pyro-Glu@N-term | cleaved S-Q@N-term | -0.0004 | 1267.677 | 2 | 14 | 1.1.1.3238.25 | Winner |

116

| Contrib | Conf | Sequence | Modifications | Cleavages | ∆Mass | Prec MW | z | Sc | Spectrum | Type |
| --- | --- | --- | --- | --- | --- | --- | --- | --- | --- | --- |
| 2 | 99 | DTGTYGFLLPER |  |  | -0.0012 | 1367.671 | 2 | 12 | 1.1.1.3662.14 | Winner |

117

| Contrib | Conf | Sequence | Modifications | Cleavages | ∆Mass | Prec MW | z | Sc | Spectrum | Type |
| --- | --- | --- | --- | --- | --- | --- | --- | --- | --- | --- |
| 2 | 99 | AMAVEDIISR |  |  | 0.0056 | 1103.57 | 2 | 10 | 1.1.1.3329.13 | Winner |

118

| Contrib | Conf | Sequence | Modifications | Cleavages | ∆Mass | Prec MW | z | Sc | Spectrum | Type |
| --- | --- | --- | --- | --- | --- | --- | --- | --- | --- | --- |
| 2 | 99 | SYELTQPPSVSVSPGQTAR |  |  | 0.0008 | 2002.997 | 3 | 13 | 1.1.1.3217.28 | Winner |

119

| Contrib | Conf | Sequence | Modifications | Cleavages | ∆Mass | Prec MW | z | Sc | Spectrum | Type |
| --- | --- | --- | --- | --- | --- | --- | --- | --- | --- | --- |
| 2 | 99 | DIQMTQSPSTLSASVGDR |  | cleaved C-D@N-term | -0.0066 | 1891.888 | 2 | 11 | 1.1.1.3218.45 | Winner |

120

| Contrib | Conf | Sequence | Modifications | Cleavages | ∆Mass | Prec MW | z | Sc | Spectrum | Type |
| --- | --- | --- | --- | --- | --- | --- | --- | --- | --- | --- |
| 2 | 99 | NLILAGVK |  |  | 0.0036 | 826.5313 | 2 | 9 | 1.1.1.3038.2 | Winner |

121

| Contrib | Conf | Sequence | Modifications | Cleavages | ∆Mass | Prec MW | z | Sc | Spectrum | Type |
| --- | --- | --- | --- | --- | --- | --- | --- | --- | --- | --- |
| 2 | 99 | LGKDAVEDLESVGK |  | missed K-D@3 | -0.0043 | 1458.752 | 3 | 17 | 1.1.1.3236.5 | Winner |

122

| Contrib | Conf | Sequence | Modifications | Cleavages | ∆Mass | Prec MW | z | Sc | Spectrum | Type |
| --- | --- | --- | --- | --- | --- | --- | --- | --- | --- | --- |
| 2 | 99 | ALDFAVGEYNK |  |  | 0.0007 | 1225.599 | 2 | 12 | 1.1.1.3326.23 | Winner |

123

| Contrib | Conf | Sequence | Modifications | Cleavages | ∆Mass | Prec MW | z | Sc | Spectrum | Type |
| --- | --- | --- | --- | --- | --- | --- | --- | --- | --- | --- |
| 1.82 | 99 | IPACIAGER | Carbamidomethyl(C)@4 |  | -0.0029 | 985.4987 | 2 | 12 | 1.1.1.2650.13 | Winner |

124

| Contrib | Conf | Sequence | Modifications | Cleavages | ∆Mass | Prec MW | z | Sc | Spectrum | Type |
| --- | --- | --- | --- | --- | --- | --- | --- | --- | --- | --- |
| 1.82 | 99 | EIMENYNIALR |  |  | 0.0087 | 1364.684 | 2 | 12 | 1.1.1.3380.11 | Winner |

FC-TSP (PEG4000)-gel-rep 1.1

| Contrib | Conf | Sequence | Modifications | Cleavages | ∆Mass | Prec MW | z | Sc | Spectrum | Type |
| --- | --- | --- | --- | --- | --- | --- | --- | --- | --- | --- |
| 2 | 99 | KGHVSFKPSLDQQR |  | missed K-G@1 | -0.0001 | 1625.864 | 4 | 14 | 1.1.1.2973.3 | Winner |

52

53

| Contrib | Conf | Sequence | Modifications | Cleavages | ∆Mass | Prec MW | z | Sc | Spectrum | Type |
| --- | --- | --- | --- | --- | --- | --- | --- | --- | --- | --- |
| 2 | 99 | DALSSVQESQVAQQAR |  |  | 0.0045 | 1715.848 | 3 | 17 | 1.1.1.3192.12 | Winner |

54

| Contrib | Conf | Sequence | Modifications | Cleavages | ∆Mass | Prec MW | z | Sc | Spectrum | Type |
| --- | --- | --- | --- | --- | --- | --- | --- | --- | --- | --- |
| 2 | 99 | LSSGLVTAALYGR |  |  | 0.0008 | 1306.725 | 2 | 19 | 1.1.1.3467.7 | Winner |

55

| Contrib | Conf | Sequence | Modifications | Cleavages | ∆Mass | Prec MW | z | Sc | Spectrum | Type |
| --- | --- | --- | --- | --- | --- | --- | --- | --- | --- | --- |
| 2 | 99 | VDNALQSGNSQESVTEQDSK |  |  | 1.9744 | 2136.936 | 3 | 16 | 1.1.1.3022.24 | Winner |

57

| Contrib | Conf | Sequence | Modifications | Cleavages | ∆Mass | Prec MW | z | Sc | Spectrum | Type |
| --- | --- | --- | --- | --- | --- | --- | --- | --- | --- | --- |
| 2 | 99 | EAYPGDVFYLHSR |  |  | 0.0056 | 1552.737 | 3 | 10 | 1.1.1.3367.5 | Winner |

58

| Contrib | Conf | Sequence | Modifications | Cleavages | ∆Mass | Prec MW | z | Sc | Spectrum | Type |
| --- | --- | --- | --- | --- | --- | --- | --- | --- | --- | --- |
| 2 | 99 | TFYEPGEEITYSCKPGYVSR | Carbamidomethyl(C)@13 |  | 0.0044 | 2382.088 | 3 | 16 | 1.1.1.3325.26 | Winner |

59

| Contrib | Conf | Sequence | Modifications | Cleavages | ∆Mass | Prec MW | z | Sc | Spectrum | Type |
| --- | --- | --- | --- | --- | --- | --- | --- | --- | --- | --- |
| 2 | 99 | AFATDSTDAEEDK |  |  | 0.0117 | 1398.59 | 2 | 15 | 1.1.1.3014.5 | Winner |

60

| Contrib | Conf | Sequence | Modifications | Cleavages | ∆Mass | Prec MW | z | Sc | Spectrum | Type |
| --- | --- | --- | --- | --- | --- | --- | --- | --- | --- | --- |
| 2 | 99 | DAVEDLESVGK |  |  | 0.0004 | 1160.557 | 2 | 16 | 1.1.1.3311.7 | Winner |

61

| Contrib | Conf | Sequence | Modifications | Cleavages | ∆Mass | Prec MW | z | Sc | Spectrum | Type |
| --- | --- | --- | --- | --- | --- | --- | --- | --- | --- | --- |
| 2 | 99 | VGAHAGEYGAEALER |  |  | -0.0029 | 1528.724 | 3 | 13 | 1.1.1.3084.13 | Winner |

62

| Contrib | Conf | Sequence | Modifications | Cleavages | ∆Mass | Prec MW | z | Sc | Spectrum | Type |
| --- | --- | --- | --- | --- | --- | --- | --- | --- | --- | --- |
| 1.82 | 99 | LPLPTIK |  | cleaved F-L@N-term | 0.0084 | 780.5193 | 2 | 7 | 1.1.1.3317.3 | Winner |

63

| Contrib | Conf | Sequence | Modifications | Cleavages | ∆Mass | Prec MW | z | Sc | Spectrum | Type |
| --- | --- | --- | --- | --- | --- | --- | --- | --- | --- | --- |
| 1.72 | 99 | NILTSNNIDVK |  |  | 0.0023 | 1229.664 | 2 | 11 | 1.1.1.3247.16 | Winner |

64

| Contrib | Conf | Sequence | Modifications | Cleavages | ∆Mass | Prec MW | z | Sc | Spectrum | Type |
| --- | --- | --- | --- | --- | --- | --- | --- | --- | --- | --- |
| 1.49 | 99 | LLNEKNISLTK | Deamidated(N)@6 | missed K-N@5 | 0.0121 | 1272.741 | 3 | 9 | 1.1.1.3185.9 | Winner |

FC-TSP (PEG4000)-gel-rep 2.1

59

| Contrib | Conf | Sequence | Modifications | Cleavages | ∆Mass | Prec MW | z | Sc | Spectrum | Type |
| --- | --- | --- | --- | --- | --- | --- | --- | --- | --- | --- |
| 2 | 99 | NALALFVLPK |  |  | 0.0004 | 1084.665 | 2 | 14 | 1.1.1.2565.4 | Winner |

60

| Contrib | Conf | Sequence | Modifications | Cleavages | ∆Mass | Prec MW | z | Sc | Spectrum | Type |
| --- | --- | --- | --- | --- | --- | --- | --- | --- | --- | --- |
| 2 | 99 | VLGAFSDGLAHLDNLK |  |  | -0.0151 | 1668.869 | 3 | 13 | 1.1.1.2459.6 | Winner |

61．

| Contrib | Conf | Sequence | Modifications | Cleavages | ∆Mass | Prec MW | z | Sc | Spectrum | Type |
| --- | --- | --- | --- | --- | --- | --- | --- | --- | --- | --- |
| 2 | 99 | VGAHAGEYGAEALER |  |  | 0.0007 | 1528.728 | 3 | 17 | 1.1.1.1995.19 | Winner |

63

| Contrib | Conf | Sequence | Modifications | Cleavages | ∆Mass | Prec MW | z | Sc | Spectrum | Type |
| --- | --- | --- | --- | --- | --- | --- | --- | --- | --- | --- |
| 2 | 99 | ENAGEDPGLAR |  |  | -0.0015 | 1127.519 | 2 | 14 | 1.1.1.1834.33 | Winner |

65

| Contrib | Conf | Sequence | Modifications | Cleavages | ∆Mass | Prec MW | z | Sc | Spectrum | Type |
| --- | --- | --- | --- | --- | --- | --- | --- | --- | --- | --- |
| 1.77 | 99 | NILTSNNIDVK |  |  | 0.0034 | 1229.665 | 2 | 14 | 1.1.1.2157.7 | Winner |

66

| Contrib | Conf | Sequence | Modifications | Cleavages | ∆Mass | Prec MW | z | Sc | Spectrum | Type |
| --- | --- | --- | --- | --- | --- | --- | --- | --- | --- | --- |
| 1.32 | 99 | QRQEELCLAR | Carbamidomethyl(C)@7 | missed R-Q@2 | -0.0015 | 1301.649 | 3 | 8 | 1.1.1.1923.14 | Winner |

67

| Contrib | Conf | Sequence | Modifications | Cleavages | ∆Mass | Prec MW | z | Sc | Spectrum | Type |
| --- | --- | --- | --- | --- | --- | --- | --- | --- | --- | --- |
| 1.41 | 99 | AFATDSTDAEEDK |  |  | 0.0108 | 1398.59 | 2 | 13 | 1.1.1.1926.12 | Winner |

FC-TSP (PEG4000)-gel-rep 1.2

51

| Contrib | Conf | Sequence | Modifications | Cleavages | ∆Mass | Prec MW | z | Sc | Spectrum | Type |
| --- | --- | --- | --- | --- | --- | --- | --- | --- | --- | --- |
| 2 | 99 | ALDNLAR |  |  | -0.0024 | 771.4214 | 2 | 10 | 1.1.1.3522.3 | Winner |

52

| Contrib | Conf | Sequence | Modifications | Cleavages | ∆Mass | Prec MW | z | Sc | Spectrum | Type |
| --- | --- | --- | --- | --- | --- | --- | --- | --- | --- | --- |
| 2 | 99 | ENAGEDPGLAR |  |  | -0.0024 | 1127.518 | 2 | 11 | 1.1.1.3440.21 | Winner |

FC-TSP (PEG4000)-gel-rep 2.2

45

| Contrib | Conf | Sequence | Modifications | Cleavages | ∆Mass | Prec MW | z | Sc | Spectrum | Type |
| --- | --- | --- | --- | --- | --- | --- | --- | --- | --- | --- |
| 2 | 99 | AGEVQEPELR |  |  | -0.0037 | 1126.558 | 2 | 13 | 1.1.1.2250.9 | Winner |

46

| Contrib | Conf | Sequence | Modifications | Cleavages | ∆Mass | Prec MW | z | Sc | Spectrum | Type |
| --- | --- | --- | --- | --- | --- | --- | --- | --- | --- | --- |
| 2 | 99 | ALDNLAR |  |  | -0.0021 | 771.4219 | 2 | 9 | 1.1.1.2187.11 | Winner |

47

| Contrib | Conf | Sequence | Modifications | Cleavages | ∆Mass | Prec MW | z | Sc | Spectrum | Type |
| --- | --- | --- | --- | --- | --- | --- | --- | --- | --- | --- |
| 2 | 99 | ASAGLLGAHAAAITAYALTLTK |  |  | 0.0018 | 2084.165 | 3 | 26 | 1.1.1.2889.7 | Winner |

48

| Contrib | Conf | Sequence | Modifications | Cleavages | ∆Mass | Prec MW | z | Sc | Spectrum | Type |
| --- | --- | --- | --- | --- | --- | --- | --- | --- | --- | --- |
| 2 | 99 | DALSSVQESQVAQQAR |  |  | 0.0025 | 1715.847 | 3 | 10 | 1.1.1.2394.23 | Winner |

49

| Contrib | Conf | Sequence | Modifications | Cleavages | ∆Mass | Prec MW | z | Sc | Spectrum | Type |
| --- | --- | --- | --- | --- | --- | --- | --- | --- | --- | --- |
| 1.72 | 99 | AATVGSLAGQPLQER |  |  | 0.0069 | 1496.802 | 2 | 9 | 1.1.1.2407.35 | Winner |

50

| Contrib | Conf | Sequence | Modifications | Cleavages | ∆Mass | Prec MW | z | Sc | Spectrum | Type |
| --- | --- | --- | --- | --- | --- | --- | --- | --- | --- | --- |
| 1.49 | 99 | VAAGAFQGLR |  |  | -0.0007 | 988.5447 | 2 | 9 | 1.1.1.2393.21 | Winner |

51

| Contrib | Conf | Sequence | Modifications | Cleavages | ∆Mass | Prec MW | z | Sc | Spectrum | Type |
| --- | --- | --- | --- | --- | --- | --- | --- | --- | --- | --- |
| 1.36 | 99 | LAAAVSNFGYDLYR |  |  | -0.0039 | 1558.774 | 2 | 14 | 1.1.1.2706.27 | Winner |

FC-TSP (PEG6000)-gel-rep 1.1

82

| Contrib | Conf | Sequence | Modifications | Cleavages | ∆Mass | Prec MW | z | Sc | Spectrum | Type |
| --- | --- | --- | --- | --- | --- | --- | --- | --- | --- | --- |
| 2 | 99 | VTEPISAESGEQVER |  |  | 0.0002 | 1629.785 | 2 | 14 | 1.1.1.1838.36 | Winner |

84

| Contrib | Conf | Sequence | Modifications | Cleavages | ∆Mass | Prec MW | z | Sc | Spectrum | Type |
| --- | --- | --- | --- | --- | --- | --- | --- | --- | --- | --- |
| 2 | 99 | IAYGTQGSSGYSLR |  |  | 0.0002 | 1458.71 | 2 | 15 | 1.1.1.1928.36 | Winner |

85

| Contrib | Conf | Sequence | Modifications | Cleavages | ∆Mass | Prec MW | z | Sc | Spectrum | Type |
| --- | --- | --- | --- | --- | --- | --- | --- | --- | --- | --- |
| 2 | 99 | VVLHPNYHQVDIGLIK |  |  | 0.0023 | 1844.033 | 4 | 10 | 1.1.1.2086.6 | Winner |

87

| Contrib | Conf | Sequence | Modifications | Cleavages | ∆Mass | Prec MW | z | Sc | Spectrum | Type |
| --- | --- | --- | --- | --- | --- | --- | --- | --- | --- | --- |
| 2 | 99 | EAYPGDVFYLHSR |  |  | -0.0003 | 1552.731 | 3 | 10 | 1.1.1.2123.9 | Winner |

88

| Contrib | Conf | Sequence | Modifications | Cleavages | ∆Mass | Prec MW | z | Sc | Spectrum | Type |
| --- | --- | --- | --- | --- | --- | --- | --- | --- | --- | --- |
| 2 | 99 | DASGATFTWTPSSGK |  |  | 0.0027 | 1511.692 | 2 | 20 | 1.1.1.2059.15 | Winner |

89

| Contrib | Conf | Sequence | Modifications | Cleavages | ∆Mass | Prec MW | z | Sc | Spectrum | Type |
| --- | --- | --- | --- | --- | --- | --- | --- | --- | --- | --- |
| 2 | 99 | EVQLLESGGGLVQPGGSLR |  | cleaved C-E@N-term | -0.0006 | 1895.011 | 2 | 17 | 1.1.1.2225.36 | Winner |

90

| Contrib | Conf | Sequence | Modifications | Cleavages | ∆Mass | Prec MW | z | Sc | Spectrum | Type |
| --- | --- | --- | --- | --- | --- | --- | --- | --- | --- | --- |
| 2 | 99 | LPLPTIK |  | cleaved F-L@N-term | 0.0101 | 780.5211 | 2 | 7 | 1.1.1.2070.2 | Winner |

91

| Contrib | Conf | Sequence | Modifications | Cleavages | ∆Mass | Prec MW | z | Sc | Spectrum | Type |
| --- | --- | --- | --- | --- | --- | --- | --- | --- | --- | --- |
| 2 | 99 | GLVVPVIR |  |  | -0.0008 | 851.5585 | 2 | 7 | 1.1.1.2146.2 | Winner |

92

| Contrib | Conf | Sequence | Modifications | Cleavages | ∆Mass | Prec MW | z | Sc | Spectrum | Type |
| --- | --- | --- | --- | --- | --- | --- | --- | --- | --- | --- |
| 2 | 99 | VLENAEGAR |  |  | -0.0047 | 957.4833 | 2 | 13 | 1.1.1.1588.4 | Winner |

93

| Contrib | Conf | Sequence | Modifications | Cleavages | ∆Mass | Prec MW | z | Sc | Spectrum | Type |
| --- | --- | --- | --- | --- | --- | --- | --- | --- | --- | --- |
| 2 | 99 | QVQLVQSGAEVK | Deamidated(Q)@1 | cleaved S-Q@N-term | -0.0015 | 1285.686 | 2 | 18 | 1.1.1.1854.23 | Winner |

94

| Contrib | Conf | Sequence | Modifications | Cleavages | ∆Mass | Prec MW | z | Sc | Spectrum | Type |
| --- | --- | --- | --- | --- | --- | --- | --- | --- | --- | --- |
| 2 | 99 | FSGSGSGTDFTLK |  |  | 0.0039 | 1302.613 | 2 | 15 | 1.1.1.1984.10 | Winner |

95

| Contrib | Conf | Sequence | Modifications | Cleavages | ∆Mass | Prec MW | z | Sc | Spectrum | Type |
| --- | --- | --- | --- | --- | --- | --- | --- | --- | --- | --- |
| 2 | 99 | VAVFGAGGVGK |  |  | 0.0007 | 960.5401 | 2 | 11 | 1.1.1.2031.20 | Winner |

FC-TSP (PEG6000)-gel-rep 2.1

78

| Contrib | Conf | Sequence | Modifications | Cleavages | ∆Mass | Prec MW | z | Sc | Spectrum | Type |
| --- | --- | --- | --- | --- | --- | --- | --- | --- | --- | --- |
| 1.92 | 99 | ATVVYQGER |  |  | -0.0006 | 1021.519 | 2 | 14 | 1.1.1.1821.10 | Winner |

79

| Contrib | Conf | Sequence | Modifications | Cleavages | ∆Mass | Prec MW | z | Sc | Spectrum | Type |
| --- | --- | --- | --- | --- | --- | --- | --- | --- | --- | --- |
| 2 | 99 | EVQLLESGGGLVQPGGSLR |  | cleaved C-E@N-term | 0.0036 | 1895.015 | 2 | 23 | 1.1.1.2353.23 | Winner |

80

| Contrib | Conf | Sequence | Modifications | Cleavages | ∆Mass | Prec MW | z | Sc | Spectrum | Type |
| --- | --- | --- | --- | --- | --- | --- | --- | --- | --- | --- |
| 2 | 99 | DASGATFTWTPSSGK |  |  | 0.0028 | 1511.692 | 2 | 19 | 1.1.1.2197.20 | Winner |

81

| Contrib | Conf | Sequence | Modifications | Cleavages | ∆Mass | Prec MW | z | Sc | Spectrum | Type |
| --- | --- | --- | --- | --- | --- | --- | --- | --- | --- | --- |
| 2 | 99 | AAPSVTLFPPSSEELQANK |  |  | 0.001 | 1985.012 | 2 | 19 | 1.1.1.2321.15 | Winner |

82

| Contrib | Conf | Sequence | Modifications | Cleavages | ∆Mass | Prec MW | z | Sc | Spectrum | Type |
| --- | --- | --- | --- | --- | --- | --- | --- | --- | --- | --- |
| 2 | 99 | SHLIIAQVAK |  |  | -0.0016 | 1078.648 | 3 | 11 | 1.1.1.1978.2 | Winner |

83

| Contrib | Conf | Sequence | Modifications | Cleavages | ∆Mass | Prec MW | z | Sc | Spectrum | Type |
| --- | --- | --- | --- | --- | --- | --- | --- | --- | --- | --- |
| 2 | 99 | QVQLVQSGAEVK | Deamidated(Q)@1 | cleaved S-Q@N-term | -0.0011 | 1285.687 | 2 | 19 | 1.1.1.1997.19 | Winner |

84

| Contrib | Conf | Sequence | Modifications | Cleavages | ∆Mass | Prec MW | z | Sc | Spectrum | Type |
| --- | --- | --- | --- | --- | --- | --- | --- | --- | --- | --- |
| 2 | 99 | FSGSGSGTDFTLK |  |  | 0 | 1302.609 | 2 | 18 | 1.1.1.2107.12 | Winner |

85

| Contrib | Conf | Sequence | Modifications | Cleavages | ∆Mass | Prec MW | z | Sc | Spectrum | Type |
| --- | --- | --- | --- | --- | --- | --- | --- | --- | --- | --- |
| 2 | 99 | DALSSVQESQVAQQAR |  |  | -0.0002 | 1715.844 | 2 | 14 | 1.1.1.2073.35 | Winner |

86

| Contrib | Conf | Sequence | Modifications | Cleavages | ∆Mass | Prec MW | z | Sc | Spectrum | Type |
| --- | --- | --- | --- | --- | --- | --- | --- | --- | --- | --- |
| 2 | 99 | QVQLVESGGGVVQPGR | Gln->pyro-Glu@N-term |  | 0.0024 | 1591.834 | 2 | 17 | 1.1.1.2297.11 | Winner |

90

| Contrib | Conf | Sequence | Modifications | Cleavages | ∆Mass | Prec MW | z | Sc | Spectrum | Type |
| --- | --- | --- | --- | --- | --- | --- | --- | --- | --- | --- |
| 1.37 | 99 | GLVVPVIR |  |  | -0.0026 | 851.5567 | 2 | 9 | 1.1.1.2278.2 | Winner |

FC-TSP (PEG6000)-gel-rep 1.2

23

| Contrib | Conf | Sequence | Modifications | Cleavages | ∆Mass | Prec MW | z | Sc | Spectrum | Type |
| --- | --- | --- | --- | --- | --- | --- | --- | --- | --- | --- |
| 2 | 99 | ATVVYQGER |  |  | -0.0012 | 1021.518 | 2 | 12 | 1.1.1.2072.10 | Winner |

24

| Contrib | Conf | Sequence | Modifications | Cleavages | ∆Mass | Prec MW | z | Sc | Spectrum | Type |
| --- | --- | --- | --- | --- | --- | --- | --- | --- | --- | --- |
| 2 | 99 | AFATDSTDAEEDK |  |  | 0.0124 | 1398.591 | 2 | 12 | 1.1.1.2137.35 | Winner |

25

| Contrib | Conf | Sequence | Modifications | Cleavages | ∆Mass | Prec MW | z | Sc | Spectrum | Type |
| --- | --- | --- | --- | --- | --- | --- | --- | --- | --- | --- |
| 2 | 99 | LNILNAK |  |  | -0.0052 | 784.4755 | 2 | 7 | 1.1.1.2288.5 | Winner |

FC-TSP (PEG6000)-gel-rep 2.2

18

| Contrib | Conf | Sequence | Modifications | Cleavages | ∆Mass | Prec MW | z | Sc | Spectrum | Type |
| --- | --- | --- | --- | --- | --- | --- | --- | --- | --- | --- |
| 2 | 99 | LGNQEPGGQTALK |  |  | 0.0015 | 1311.68 | 2 | 13 | 1.1.1.2015.33 | Winner |

19

| Contrib | Conf | Sequence | Modifications | Cleavages | ∆Mass | Prec MW | z | Sc | Spectrum | Type |
| --- | --- | --- | --- | --- | --- | --- | --- | --- | --- | --- |
| 2 | 99 | DASGVTFTWTPSSGK |  |  | -0.0058 | 1539.715 | 2 | 18 | 1.1.1.2447.17 | Winner |

20

| Contrib | Conf | Sequence | Modifications | Cleavages | ∆Mass | Prec MW | z | Sc | Spectrum | Type |
| --- | --- | --- | --- | --- | --- | --- | --- | --- | --- | --- |
| 2 | 99 | GTWTQPFDLASTR |  |  | -0.0043 | 1478.711 | 2 | 13 | 1.1.1.2541.28 | Winner |

21

| Contrib | Conf | Sequence | Modifications | Cleavages | ∆Mass | Prec MW | z | Sc | Spectrum | Type |
| --- | --- | --- | --- | --- | --- | --- | --- | --- | --- | --- |
| 1.85 | 99 | KEAGLLAAVTLTQK | Deamidated(Q)@13 | missed K-E@1 | -0.0113 | 1442.823 | 2 | 11 | 1.1.1.2680.16 | Winner |

22

| Contrib | Conf | Sequence | Modifications | Cleavages | ∆Mass | Prec MW | z | Sc | Spectrum | Type |
| --- | --- | --- | --- | --- | --- | --- | --- | --- | --- | --- |
| 1.54 | 99 | DASGATFTWTPSSGK |  |  | 0.0049 | 1511.694 | 2 | 15 | 1.1.1.2359.28 | Winner |
